# Extreme risk spillovers between US and Chinese agricultural futures markets in crises: A dependence-switching copula-CoVaR model

**DOI:** 10.1371/journal.pone.0299237

**Published:** 2024-03-06

**Authors:** Xin Hu, Bo Zhu, Bokai Zhang, Lidan Zeng

**Affiliations:** School of Finance and Institute of Chinese Financial Studies, Southwestern University of Finance and Economics, Chengdu, China; University of Barcelona: Universitat de Barcelona, SPAIN

## Abstract

The linkages between the US and China, the world’s two major agricultural powers, have brought great uncertainty to the global food markets. Inspired by these, this paper examines the extreme risk spillovers between US and Chinese agricultural futures markets during significant crises. We use a copula-conditional value at risk (CoVaR) model with Markov-switching regimes to capture the tail dependence in their pair markets. The study covers the period from January 2006 to December 2022 and identifies two distinct dependence regimes (stable and crisis periods). Moreover, we find significant and asymmetric upside/downside extreme risk spillovers between the US and Chinese markets, which are highly volatile in crises. Additionally, the impact of international capital flows (the financial channel) on risk spillovers is particularly pronounced during the global financial crisis. During the period of the COVID-19 pandemic and the Russia-Ukraine 2022 war, the impact of supply chain disruptions (the non-financial channel) is highlighted. Our findings provide a theoretical reference for monitoring the co-movements in agricultural futures markets and practical insights for managing investment portfolios and enhancing food market stability during crises.

## 1. Introduction

Recently, global agricultural/food markets have become increasingly volatile due to frequent and severe economic and socio-political shocks, such as the global financial crisis (GFC), weather extremes, trade wars, the COVID-19 pandemic, and geopolitical conflicts. The agricultural futures market is particularly susceptible to these extreme shocks, and the consequent excess volatility will spill into spot markets [1]. Notably, the COVID-19 pandemic in 2020 and the Russia-Ukraine war in 2022 further expose the interconnectedness among global agricultural markets and the vulnerability of the global food system [2–4]. Due to economic globalization, risk event shocks in one country can quickly spread to others along their economic linkages. They may even trigger systemic risk contagion in global agricultural futures markets, which sparks great concerns about food security. Thus, investigating extreme risk spillovers across agricultural futures markets, especially during crises, is highly intriguing.

The US and China have a pivotal role in the global agricultural futures markets, and the stability of their markets is a concern for international regulators and investors. Focusing on the extreme risk spillover effects between these two markets is typical for two main reasons: First, China and the US, the world’s two biggest consumers/producers of agricultural commodities, have close economic and trade relations. For instance, according to USA Trade Online, in 2022, the US exported 30.1 billion US dollars of agricultural products to China (soybean exports accounted for about 60%). Second, the deepening financialization of agricultural commodities and the integration of global markets have led to closer ties in their agricultural futures markets. Furthermore, we provide an example of their co-movements: during the GFC, the prices of US agricultural futures surged, and China also experienced a significant price rise despite implementing various intervention policies.

In recent years, cross-border risk spillovers in the international agricultural futures markets have attracted much attention [5]. Previous literature extensively estimates the co-movements between the US and Chinese agricultural futures markets [6–10]. Most of them discuss the risk spillovers between these two markets using volatility and confirm the existence of co-movements. However, volatility spillovers fail to portray the risk spillovers under extreme market conditions and ignore the asymmetry of upside and downside distress. The extreme risk spillover effects caused by significant crises are still underexplored, let alone the differences in the interdependence between stable and crisis periods. Since markets are usually more closely linked in times of crisis than in times of stability [11, 12], it is imperative to fill this gap by studying the extreme risk spillovers between the two agricultural powers and incorporating the changes in dependence structure in turbulent times.

Our work proceeds in two steps. First, using the daily prices of agricultural commodity futures in the two countries from January 10, 2006, to December 31, 2022, and employing a Markov regime-switching copula-conditional value at risk (CoVaR) model, we measure the extreme risk spillover effects. The analyzed extreme risk spillovers focus on the fluctuations under extreme market conditions and depict the “net” negative externalities, excluding the dependence derived from the common factors. Furthermore, it is different from the conventional copula-CoVaR model by Reboredo and Ugolini [13], Mensi et al. [14], and Wang and Xu [15]. We introduce a hidden two-state Markov chain for the intercept term of the time-varying dependence parameter in line with da Silva Filho et al. [16], Ji et al. [17], and Jiao and Ye [18]. In this way, the interdependence between “stable” (low dependence) and “crisis” (high dependence) regimes is distinguished. Also, it is different from the switching between upside and downside dependence regimes by Ji et al. [19]. Second, we explore the driving factors of extreme risk spillovers, especially during the GFC (August 2007–April 2009), the US-China trade war (January 2018 to December 2022), the COVID-19 pandemic (December 2019 to December 2022), and the Russia-Ukraine war (March 2022 to December 2022).

The contributions of this study can be summarized as follows: First, our empirical analysis enriches studies on the information transmission between the US and Chinese markets by portraying the tail risk spillovers under extreme event shocks. Previous studies focus on information transmission regarding return and volatility, but few analyze the extreme risk spillovers. Hence, this paper serves as a supplement in this respect. Second, by distinguishing the low and high dependence regimes, our study provides empirical evidence for the changes in the dependence structure between the two markets in a stable environment and a turbulent period. This highlights the vulnerability of interconnected food markets in crises using the theory of dependence-switching regimes. Third, this analysis sheds light on the driving factors of extreme risk spillover effects during recent crises. During the period of the COVID-19 pandemic and the Russia-Ukraine 2022 war, it not only highlights the impact of international capital flows (the financial channel) but also emphasizes trade and production shocks (the non-financial channel). Our work outlines important implications for investors to manage their portfolios and for policymakers to improve the stability of food markets.

The rest of this paper is organized as follows: Section 2 briefly reviews the previous literature on risk spillovers in commodities markets as well as the correlations between US and Chinese agricultural markets. The Markov regime-switching copula-CoVaR model used to measure the extreme risk spillovers is introduced in Section 3. Section 4 details the data and stylized facts of the futures prices. Section 5 presents the empirical findings on risk spillovers and investigates their influencing factors during different crises. Finally, Section 6 summarizes conclusions and recommendations.

## 2. Literature review

With the financialization of the commodity market, the risk spillovers in the commodity markets have become a new key point of systemic risk contagion and have been extensively investigated in the literature. For example, Kumar et al. [20] study the correlations in the commodity markets of the Asia-Pacific region. Bouri et al. [21] analyze the volatility connectedness of commodity futures and explore its determinants, providing valuable insights into the factors driving volatility in commodity markets. Furthermore, Vardar et al. [22], Ahmed and Huo [23], Bouri et al. [24], and Zhang et al. [25] contribute to understanding the dynamics of shock propagation and volatility spillover across stock and commodity markets, offering insightful information about market dynamics and interactions.

Among the commodities, agricultural markets are increasingly active, and the linkages among agricultural markets in different countries/regions are increasingly closer [5]. For example, Hernandez et al. [26] show the volatility spillovers in major agricultural futures markets for corn, wheat, and soybeans. They find that these agricultural markets are highly interconnected, exhibiting internal and external risk contagion effects across countries. As major trading partners, the US and China hold significant positions in the agricultural commodity markets. The US markets stand as global leaders in activity, while the Chinese markets are experiencing rapid growth [27]. Therefore, the interaction between these two markets is quite important, as it can influence other markets [6].

Scholars have conducted extensive research on price information spillovers between agricultural commodity futures markets in China and the US with respect to return and volatility [6, 8, 10]. Most studies claim that the US has pricing power [28]. Some also find bidirectional spillovers between the US and China, in which the US substantially influences China more than the opposite [18, 29–31]. In addition, Liu et al. [7] and Jiang et al. [9] show that the spillovers in soybean futures markets from China to the US gradually increase. Some other studies emphasize China’s pricing power—for example, Lee et al. [32] show that China dominates information spillovers to the US in the corn and soybean markets. These studies using return and volatility fail to capture the risk contagion under extreme market conditions and treat the downside and upside co-movement equally. A few studies have shown the existence of extreme risk spillovers between these two markets [33, 34]. However, they fail to capture the structural changes in dependence in crises.

In the context of extreme shocks, the risk spillover between commodity markets attracts particular attention [11, 12, 35, 36]. Cheng et al. [37] delve into the volatility linkages between energy and agricultural commodities during the US-China trade war, underscoring commodity markets’ heightened interconnectivity and responsiveness to trade disputes. Li et al. [38] investigate how geopolitical risk, economic policy uncertainty, and climate policy uncertainty influence the commodity markets in America and China. Iqbal et al. [39] reveal the characteristics of linkages in commodity markets during crises such as COVID-19. Wang et al. [40] investigate the influence of geopolitical risk on systemic risk in commodity markets in the context of the Russia-Ukraine war. Zhu et al. [41] also point out the high volatility of commodity futures during uncertain times. These studies all highlight that the risk spillovers are likely intensified by extreme crisis events.

The research methods on risk contagion/spillover effects among financial markets mainly fall into two categories. The first consists of traditional methods such as correlation coefficients [42], co-integration tests [43], Granger-causality tests [44], vector autoregressive models [45], and generalized autoregressive conditional heteroskedasticity (GARCH) models [46]. The second comprises methods based on copula models [14, 47–53]. In comparison to the traditional methods, the second type of methods can well capture the time-varying, asymmetric, and nonlinear tail dependence structure between markets, which arises from fat tails and heteroskedasticity. However, conventional copula models do not consider the potential changes in the dependence structure during periods of financial stress. They assume that the dependence structure between financial assets is constant regardless of market conditions. In response to this limitation, Markov regime-switching copula models have been developed [16–18, 54]. These models are highlighted for their ability to eliminate the need to temporally determine the point of change in the dependence structure, as emphasized by Boubaker and Sghaier [55].

In summary, previous studies focus on the return and volatility spillovers in commodity markets (including the US and Chinese agricultural future markets). However, they have paid less attention to the tail dependence between these markets as well as the extreme/tail risk spillovers under extreme market conditions. Despite the fact that significant crisis events could trigger severe market shocks and intense regime-switching behavior, the interdependence between stable and crisis periods is still not distinguished, let alone the driving mechanisms during crises. To make an effort to fill this gap, we use the copula-CoVaR approach to capture the time-varying, asymmetric, and nonlinear characteristics of tail dependence between markets. Moreover, we apply the Markov regime-switching framework to differentiate the dependence between normal and crisis times. This model assumes that the dependence structure changes over time depending on the underlying market conditions, thus providing a more accurate estimate of risk spillovers during periods of financial stress. Lastly, we test the influencing mechanism of extreme risk spillover effects during the recent crises.

## 3. Methodology

In this section, we introduce our models in three steps. First, based on da Silva Filho et al. [16], Ji et al. [17], and Jiao and Ye [18], we develop a Markov regime-switching copula model to depict the tail dependence. Second, we measure the extreme risk spillover effects by calculating ΔCoVaR based on the chosen optimal copula functions. Third, we analyze the determinants of extreme risk spillovers based on a panel data model.

Note that the Markov-switching time-varying dependence approach proposed by da Silva Filho et al. [16] is well-justified in our study because it captures the changes in the structure of inter-market dependence well. This method adds a hidden Markov chain to the equation of the time-varying dependence parameter. As dependencies are not static but evolve over time and across different market states or regimes, incorporating the Markov-regime switching into the copula-CoVaR model can reflect the complex behavior of markets well and is crucial for understanding correlations in stressful and calm periods. In this respect, it provides a more accurate and nuanced understanding of market dynamics.

### 3.1 Markov-switching time-varying dependence modeling

#### 3.1.1 Marginal distribution

In the fitting models of the return series, we apply the auto-regressive moving average (ARMA) model to describe the mean equation and use GARCH-family models to describe the volatility equation. The ARMA (m, s)-GACRH (p, q) model is expressed as

$$r_{i,t}=\mu_{i}+\sum_{k=1}^{m}\varphi_{i,k}r_{i,t-k}+\sum_{k=1}^{s}\phi_{i,k}\epsilon_{i,t-k}$$ (1)

$$\epsilon_{i,t}=z_{i,t}\sqrt{h_{i,t}}$$ (2)

$$h_{i,t}=\omega_{i}+\sum_{k=1}^{p}\alpha {'}_{i,k}\cdot \epsilon_{i,t-k}^{2}+\sum_{k=1}^{q}\beta {'}_{i,k}\cdot h_{i,t-k}$$ (3)

where *r*_*i*,*t*_ is the return series; *ϵ*_*i*,*t*_ is the disturbance term; *z*_*i*,*t*_ is the standardized residual; *h*_*i*,*t*_ is the conditional variance; *μ*_*i*_, *φ*_*i*,*k*_, *ϕ*_*i*,*k*_, *ω*_*i*_, *α*’_*i*,*k*_, and *β*’_*i*,*k*_ are estimated parameters, satisfying *ω*_*i*_>0, *α*’_*i*,*k*_≥0, *β*’_*i*,*k*_≥0 and $0<\sum_{k=1}^{p}\alpha {'}_{i,k}+\sum_{k=1}^{q}\beta {'}_{i,k}<1$.

The optimal lags of the mean and variance equations are determined by Bayesian information criteria (BIC). In the estimation progress, we also include the extension forms of the GACRH model, including simple GARCH, exponential GARCH (EGARCH), Glosten-Jagannathan-Runkle GARCH (GJR-GARCH), integrated GARCH (IGARCH), threshold GARCH (TGARCH), absolute value GARCH (AVGARCH), nonlinear GARCH (NGARCH), nonlinear-asymmetric GARCH (NAGARCH), and asymmetric power ARCH (APARCH). Furthermore, all models allow for different marginal distributions, for example, normal, Student’s t, generalized error distribution (GED), or their skewed versions [56]. When selecting the most appropriate GARCH specification for each series, we follow an adjusted version of the BIC proposed by Antonakakis et al. [57]: $BIC_{\mathrm{adjusted}}=-2\cdot \ln(L)+k\cdot \ln(T)$, where *L* represents the maximum of the likelihood function and *k* denotes the number of insignificant parameters and the number of significant misspecification tests (including sign bias, the weighted Li-Mak test, the 1% value-at-risk (VaR) test, the 1% conditional VaR test, and the 1% duration based VaR test).

#### 3.1.2 Copula functions

The probability integral transformations of *z*_*i*,*t*_ and *z*_*j*,*t*_ are denoted by *u* and *v*, respectively. According to Sklar’s theorem, a bivariate copula used to model the tail dependence between *z*_*i*,*t*_ and *z*_*j*,*t*_ can be expressed by a copula function *C*(*u*,*v*) and the two marginal distribution functions $u=F_{z_{i}}(z_{i,t}|\theta_{1})$ and $v=F_{z_{j}}(z_{j,t}|\theta_{2})$:

$$F_{z_{i}z_{j}}(z_{i,t},z_{j,t}|F_{t-1};\theta )=C(u,v|F_{t-1};\theta_{c})=C(F_{z_{i}}(z_{i,t}|\theta_{1}),F_{z_{j}}(z_{j,t}|\theta_{2})|F_{t-1};\theta_{c})$$ (4)

Considering a time-varying copula model embedded in two-regime switching [16], Eq (4) can be rewritten as

$$\begin{array}{l} F_{z_{i}z_{j}}(z_{i,t},z_{j,t}|s_{t},F_{t-1};\theta )=\sum_{j=1}^{2}C(u,v|s_{t}=j,F_{t-1};\theta_{c})\cdot \mathrm{Pr}(s_{t}=j|F_{t-1})\\ \;=C(u,v|s_{t},F_{t-1};\theta )=C(u,v;\rho_{t,s_{t}}) \end{array}$$ (5)

where $\theta =(\theta_{1}^{\prime },\theta_{2}^{\prime },\theta_{c}^{\prime })$. The copula model specifications used to describe the tail dependence are detailed in **Table 1**.

In the copula model, the dependence parameter $\rho_{t,s_{t}}$ is subject to an ARMA (1, *q*) process [58], in which the intercept term of $\psi_{0,s_{t}}$ depends on the regime *s*_*t*_ [16]:

$$\rho_{t,s_{t}}=\Lambda (\psi_{0,s_{t}}+\psi_{1}\rho_{t-1}+\psi_{2}FV_{t})$$ (6)

where Λ(•) is the modified logistic transformation to ensure that the parameter $\rho_{t,s_{t}}$ varies in (−1, 1); *ψ*_0_, *ψ*_1_ and *ψ*_2_ represent dependence, persistence, and adjustment, respectively. Moreover, *s*_*t*_~Markov(**Prob**), and *s*_*t*_ = 0 and *s*_*t*_ = 1 assume the two states/regimes: low and high dependence, respectively. **Prob** is a 2×2 transition matrix $\left(\begin{array}{cc} Prob_{00} & Prob_{10}\\ Prob_{01} & Prob_{11} \end{array}\right)$. Then, following Ji et al. [17], we combine the low and high dependence regimes in one equation to match with Eq (6):

$$\rho_{t}=\sum_{j=1}^{2}\rho_{t,s_{t}=j}\cdot \mathrm{Pr}(s_{t}=j|F_{t})$$ (7)

**Table 1:** Copula specifications.

Copula	Function
Gaussian	$C_{N}(u,v,\rho )=\phi (\phi^{-1}(u),\phi^{-1}(v))$
Student-t	$C_{ST}(u,v,\rho ,\vartheta )=t(t_{\vartheta }^{-1}(u),t_{\vartheta }^{-1}(v))$
Gumbel	$C_{G}(u,v;\rho )=\exp\{-{\left[{\left(-\ln(u)\right)}^{\rho }+{\left(-\ln(v)\right)}^{\theta }\right]}^{1/\rho }\}$
Rotated Gumbel	$C_{RG}(u,v;\rho )=u+v-1+C_{G}(1-u,1-v;\rho )$
Clayton	$C_{CL}(u,v;\rho )=\max\{{\left(u^{-\rho }+v^{-\rho }-1\right)}^{-1/\rho },0\}$
Rotated Clayton	$C_{RCL}(u,v;\rho )=u+v-1+C_{CL}(1-u,1-v;\rho )$
Symmetrized Joe-Clayton (SJC)	For Joe-Clayton (also known as the BB7 copula), $C_{JC}(u,v|\rho_{U},\rho_{L})=1-{\left(1-{\left\{{\left[1-{\left(1-u\right)}^{\kappa }\right]}^{-\gamma }+{\left[1-{\left(1-v\right)}^{\kappa }\right]}^{-\gamma }-1\right\}}^{-1/\gamma }\right)}^{1/\kappa }$, where $\kappa =1/\log_{2}(2-\rho_{U}),\gamma =-1/\log_{2}(\rho_{L})$. So, $C_{SJC}(u,v|\rho_{U},\rho_{L})=0.5\cdot (C_{JC}(u,v|\rho_{U},\rho_{L})+C_{JC}(1-u,1-v|\rho_{U},\rho_{L})+u+v-1)$

Note: *ϕ*(⋅) is standard Gaussian distribution function; *t*(⋅) is Student t distribution function.

In the modeling of Markov-switching time-varying (MSTV) Gaussian, the forcing variable is $FV_{t}=1/q\sum_{j=1}^{q}\phi^{-1}(u_{t-j})\cdot \phi^{-1}(v_{t-j})$ where *q* is an arbitrary window length. In the MSTV Student-t copula, the quantile function *ϕ*^−1^(*x*) is substituted by $t_{\vartheta }^{-1}(x)$ with *ϑ* degrees of freedom. For the MSTV Clayton, rotated Clayton, Gumbel, and rotated Gumbel copula, $FV_{t}=1/q\sum_{j=1}^{q}\left|u_{t-j}-v_{t-j}\right|)$. For the MSTV Symmetrized Joe-Clayton (SJC) copula, we can express the upper-tail (*ρ*_*U*,*t*_) and lower-tail (*ρ*_*L*,*t*_) dependence parameters as

$$\rho_{U,t}=\Lambda (\psi_{U,0,s_{t}}+\psi_{U,1}\rho_{t-1}+\psi_{U,2}\frac{1}{q}\sum_{j=1}^{q}\left|u_{t-j}-v_{t-j}|\right)$$ (8)

$$\rho_{L,t}=\Lambda (\psi_{L,0,s_{t}}+\psi_{L,1}\rho_{t-1}+\psi_{L,2}\frac{1}{q}\sum_{j=1}^{q}\left|u_{t-j}-v_{t-j}|\right)$$ (9)

#### 3.1.3 Estimations

We utilize the inference for margins (IFM) method to estimate the parameter $\theta =(\theta_{1}^{\prime },\theta_{2}^{\prime },\theta_{c}^{\prime })$. At first, we estimate $\theta_{1}^{\prime }$ and $\theta_{2}^{\prime }$ by

$${\hat{\theta }}_{i}=\underset{\theta_{i}}{\mathrm{argmax}}\sum_{t=1}^{T}\log(f_{i,t}(z_{i,t}|F_{t-1};\theta_{i})),i=1,2$$ (10)

Next, the dependence parameters are estimated by

$${\hat{\theta }}_{c}=\underset{\theta_{c}}{\mathrm{argmax}}\sum_{t=1}^{T}\log(c_{t}({\hat{u}}_{t},{\hat{v}}_{t}|F_{t-1};\theta_{c}))$$ (11)

Under regular conditions, the IFM estimator $\hat{\theta }=({\hat{\theta }}_{1},{\hat{\theta }}_{2},{\hat{\theta }}_{c})$ is considered asymptotically normal [58]. So,

$$\sqrt{T}(\hat{\theta }-\theta )\to N(0,G^{-1}(\theta ))$$ (12)

where *G*(*θ*) is a Godambe information matrix.

### 3.2 Extreme risk spillover modeling

VaR measures the maximum loss an investor could suffer by taking a short or long position at the 1−*α* confidence level. CoVaR, introduced by Adrian and Brunnermeier [59], captures the VaR of one market condition on the distress of another market and quantifies the risk spillover effects between markets. Let $CoVaR_{\beta ,t}^{i|j}$ denote the VaR of the market *i* conditional on market *j* being under distress at time *t*. Furthermore, following Girardi and Ergün [46] and Mainik and Schaanning [60], we change the conditioning event $r_{t}^{j}=VaR_{\alpha }^{j}$ as $r_{t}^{j}\leq VaR_{\alpha }^{j}$ ($r_{t}^{j}\geq VaR_{\alpha }^{j}$) for downside (upside) risk spillovers, thus taking into account more severe distress.

Then, we can calculate the downside and upside extreme risk spillovers in US-Chinese agricultural futures markets by

$$Pr(r_{t}^{i}\leq CoVaR_{\beta ,t}^{i|j}|r_{t}^{j}\leq VaR_{\alpha ,t}^{j})=\beta$$ (13)

$$Pr(r_{t}^{i}\geq CoVaR_{\beta ,t}^{i|j}|r_{t}^{j}\geq VaR_{\alpha ,t}^{j})=\beta$$ (14)

where *α* and *β* denote the significance levels; *Pr*(•) is the probability function; $VaR_{\alpha }^{i}$ is the *α* quantile of the return series $r_{t}^{j}$. Based on the copula functions in Eq (5), Eqs (13)–(14) can be reformulated as

$$C(F_{r_{t}^{i}}(CoVaR_{\beta ,t}^{i|j}),F_{r_{t}^{j}}(VaR_{\alpha ,t}^{j}))=\alpha \beta$$ (15)

$$1-F_{r_{t}^{i}}(CoVaR_{\beta ,t}^{i|j})-F_{r_{t}^{j}}(VaR_{\alpha ,t}^{j})+C(F_{r_{t}^{i}}(CoVaR_{\beta ,t}^{i|j}),F_{r_{t}^{j}}(VaR_{\alpha ,t}^{j}))=\alpha \beta$$ (16)

where *C*(•) denotes the optimal copula function; the marginal distribution functions $F_{r_{t}^{j}}$ and $F_{r_{t}^{i}}$ are fitted by the return series $r_{t}^{j}$ and $r_{t}^{i}$, respectively. When calculating the downside CoVaR, we use *α* = *β* = 5%. When calculating the upside CoVaR, we use *α* = *β* = 95%. The 5% and 95% quantiles represent the risk levels of a portfolio commonly employed by investors and portfolio managers.

Therefore, to exclude the dependence driven by the common factors, we use $\Delta CoVaR_{\beta ,t}^{i|j}$, the difference between market *i*’s VaR conditional on market *j* being in extreme scenarios $r_{t}^{j}\leq VaR_{\alpha }^{i}$ ($r_{t}^{j}\geq VaR_{\alpha }^{i}$) and normal scenarios $r_{t}^{j}\leq VaR_{50\%}^{i}$ ($r_{t}^{j}\geq VaR_{50\%}^{i}$), to estimate the “net” risk spillovers from market *j* to market *i*. Thus, the downside/upside extreme risk spillovers from market *j* to market *i* are defined as

$$\Delta CoVaR_{\beta ,t}^{i|j}=CoVaR_{\beta ,t}^{i|j,\alpha }-CoVaR_{\beta ,t}^{i|j,50\%}$$ (17)

### 3.3 Influencing mechanism modeling

Extensive literature has revealed the driver factors of agricultural commodity price volatility, such as market fundamentals (including supply/demand shocks), trade, speculation, investor sentiment, and macro conditions [61–63]. Specifically, we use the S&P 500 Volatility Index (VIX), developed by the Chicago Board Options Exchange (CBOE), to reflect investors’ risk perceptions [64]. Economic policy uncertainty (EPU) and geopolitical risk (GPR) are also seen as important macro factors that affect the commodity markets [38, 40]. In addition, Nigatu et al. [62] and Akram [65] highlight the vital role of economic growth and exchange rates in driving fluctuations in commodity prices.

The deepening of financial internationalization has linked commodities markets across countries. Financial linkages may also be critical to risk contagion. Following Huang and Liu [66], we use the international capital inflows to study their impact on risk transmission, where the international capital includes the total financial assets, such as stocks and bonds, purchased by foreign investors. Specifically, a significant international capital inflow into agricultural commodity markets can lead to increased investment and speculation, which directly influence agricultural commodity prices. Therefore, international capital inflow is used as an indicator of financial shocks in agricultural commodity markets. Furthermore, trade globalization has strengthened economic ties between countries, thus providing a risk contagion channel for shocks [67]. This paper uses trade policy uncertainty (TPU) to serve as a proxy for trade shocks in both countries [68]. TPU reflects the level of uncertainty in tariffs, quotas, embargoes, or other trade policies, which may directly affect agricultural imports and food availability.

In addition, following Nigatu et al. [62], the changes in crop production and biofuel prices are used to reflect the supply and demand shocks in the agricultural commodity market. Specifically, the changes in crop production reflect supply fluctuations of agricultural commodities, which may affect the quantity of agricultural products available in the market. On the other hand, biofuels are often produced from agricultural raw materials, so the changes in biofuel prices may affect the demand for these agricultural products. For example, crops such as corn and oilseeds are commonly used in biofuel production; an increase in biofuel prices may lead to increased demand for these agricultural products [69].

In sum, we consider macro factors, supply, demand, trade, and financial shocks to explain the risk spillovers between the US and China. The panel regression model is constructed as

$$\Delta CoVaR_{\beta ,k,t}^{j|i}=\alpha_{0}+\theta Z+\alpha_{k}+\varphi t+u_{k,t}$$ (18)

where *α*_0_, *α*_*k*_, *φ*, and **θ** are the parameters to be estimated; *α*_*k*_, *t* and *u*_*k*,*t*_ represent individual fixed effect, time trend term, and random error term, respectively; $\Delta CoVaR_{\beta ,k,t}^{j|i}$ denotes the extreme risk spillovers from country *i* to *j* at time *t* for the commodity *k*; **Z** is a vector of explanatory variables. These regression variables are detailed in **Table 2**.

**Table 2:** Variables used in the regression analysis.

Variables	Labels	Description	
*Explained variables*:			
Extreme risk spillovers	$\Delta CoVaR_{\beta ,k,t}^{j|i}$	Spillovers from market *i* to *j* at time *t* for commodity *k*.	
*Explanatory variables*:			
Macro factors	*VIX*_*t*_	The S&P 500 volatility index, developed by the CBOE. It reflects the investors’ risk perceptions.	
	*GEPU*_*t*_	Global economic policy uncertainty index based on current-price GDP measures, developed by Davis [70]. It reflects the economic policy environment at the global level.	
	*GPR*_*t*_	Global geopolitical risk index, constructed by Caldara and Iacoviello [71]. It reflects the geopolitical environment at the global level.	
	*EXR*_*t*_	USD/RMB exchange rate.	
	*GDP*_*i*,*t*_, *GDP*_*j*,*t*_	The degree of economic development, measured by the Gross Domestic Product (GDP) Index. It is monthly and suitable for our analysis.	
Financial shocks	*Flow*_*i→j*,*t*_, *Flow*_*j→i*,*t*_	International capital inflow from country *i* (*j*) to *j* (*i*). It is in the form of the first difference of logarithms in regressions.	
Trade shocks	*TPU*_*i*,*t*_, *TPU*_*j*,*t*_	Trade policy uncertainty, developed by Davis et al. [72] and Baker et al. [73].	
Supply-side shocks	$Pduc_{i_{k},t}$, $Pduc_{j_{k},t}$	The natural logarithm of crop production in market *i* (*j*) for each commodity.	
Demand-side shocks	*Biofuel*_*t*_	The natural logarithm of biofuel prices, measured by the S&P Biofuels Spot Index.	

## 4. Data and stylized facts

### 4.1 Data

This study uses the daily closing prices of continuous futures contracts from January 10, 2006, to December 31, 2022. We consider three types of agricultural commodities that play an essential role in the food system: grain (soybean, wheat, and corn), sugar, and soybean oilseeds (soybean oil and soybean meal). Grain is recognized as the food source for millions of people. Sugar is the main sweetener in food and beverages. Soybean oilseed is a source of valued protein in livestock production, as well as a source of vegetable oil for human food. Note that rice is also the focus of global food markets. However, the rice futures in the Chinese market are listed late and less active, so they are not included in our analysis. Our final sample includes commodities with large trading volumes and significant influence in China and the US.

Sugar and soybean oil futures in the Chinese markets started trading on January 6, 2006, and January 9, 2006, respectively. Due to the unavailability of the data, this paper selects January 10, 2006, as the starting point of the sample. The prices of soybean, corn, soybean meal, and soybean oil futures are downloaded from the Dalian Commodity Exchange (DCE, China) and the Chicago Board of Trade (CBOT, US). The prices of sugar futures are downloaded from the Zhengzhou Commodity Exchange (ZCE, China) and the Intercontinental Exchange (ICE, US). The prices of wheat futures come from ZCE and CBOT.

**Fig 1:**
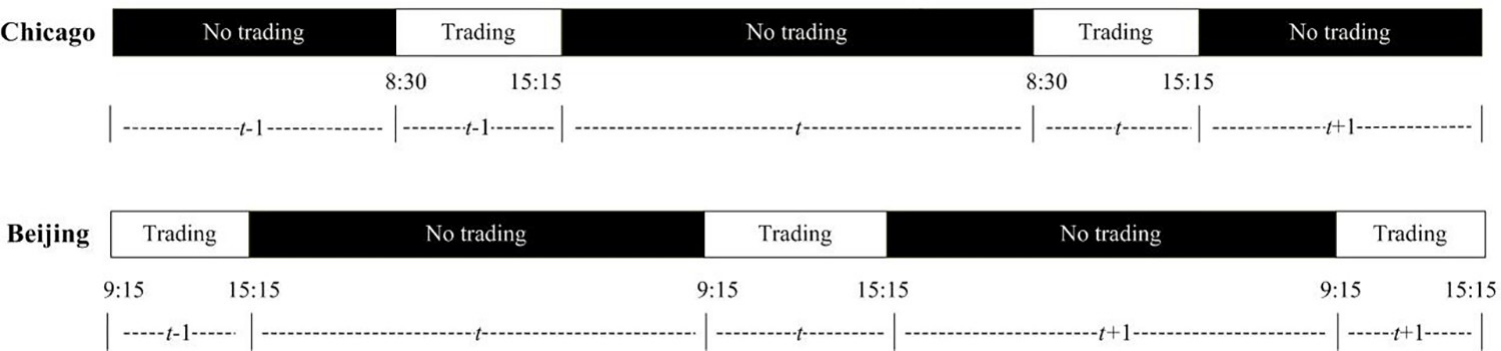
Trading hours in the US and China.

Before conducting the analysis, we adjust the currency unit of all Chinese futures prices by US dollars to ensure comparability between the two countries. Moreover, we use the trading schedule of the DCE as the benchmark. All missing values of the futures prices are filled with the prices of the previous trading day. The log return *R*_*t*_ is calculated by $R_{t}=\ln(P_{t}/P_{t-1})$, where *P*_*t*_ is the closing price of futures. Here, *t* is the local time for the US and China. Note that Beijing is about 13 hours earlier than Chicago, and trading and non-trading time in China is nearly the opposite of the US (see **Fig 1**). Therefore, estimations of risk spillovers from China to the US are based on $R_{t}^{i,China}$ and $R_{t}^{i,US}$, and risk spillovers from the US to China are based on $R_{t+1}^{i,China}$ and $R_{t}^{i,US}$.

In the section on regression analysis, we use monthly observations of mechanism variables. The data on biofuel prices and VIX are obtained from the Bloomberg database. The data on the market supply, the GDP index, the exchange rate, and international investment are taken from the WIND Financial database. The data on GEPU, GPR, and TPU are available at https://www.policyuncertainty.com.html. To build a regression, we average the extreme risk spillovers by month. The final sample for regression includes 204 months and six commodities, totaling 1224 observations. Any missing values for crop production are filled with the values from the previous month.

### 4.2 Stylized facts

**Fig 2:**
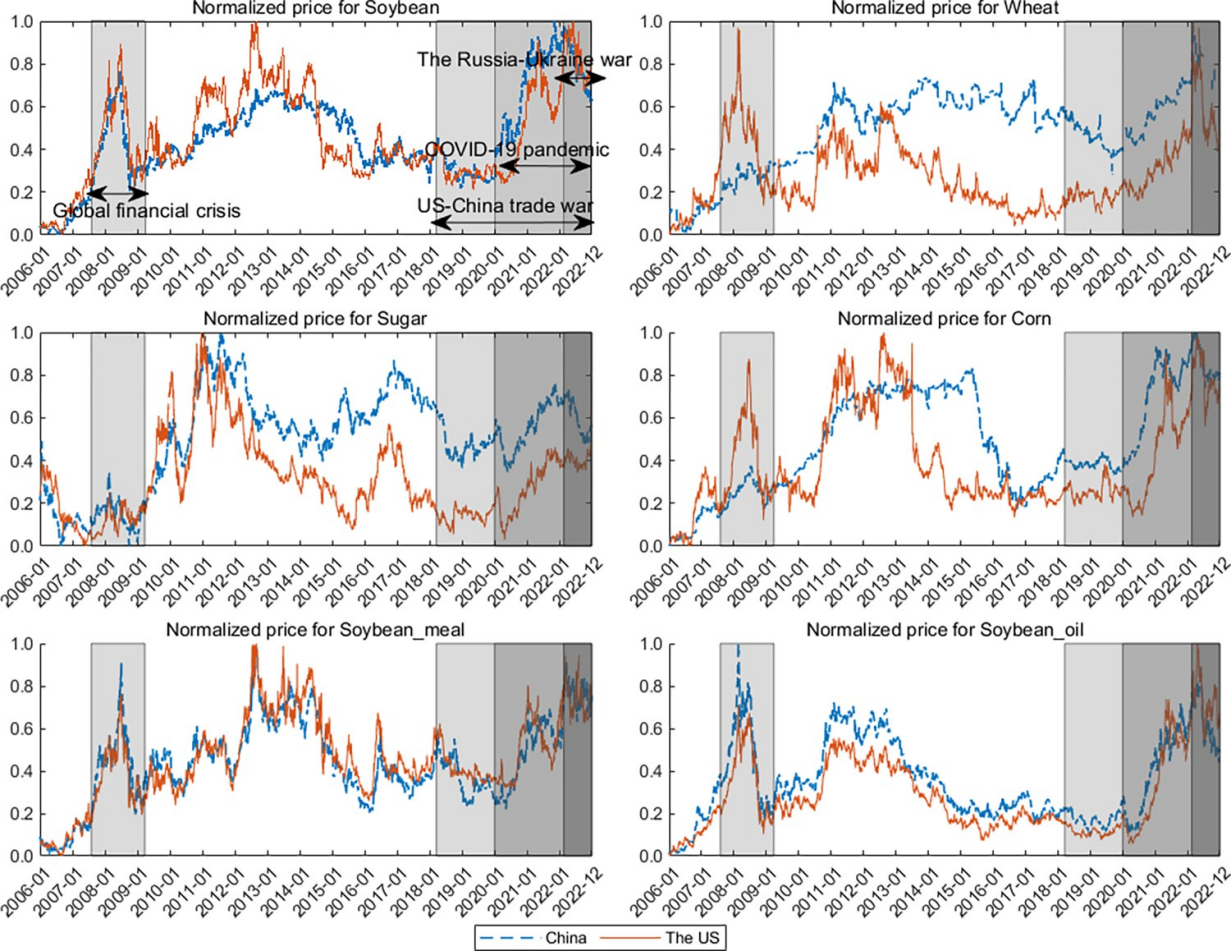
Dynamics of US-Chinese agricultural futures prices. In the figure, the solid red line represents the dynamic trend of futures prices in the US, while the blue dashed line represents the dynamic of futures prices in China. The shaded areas correspond to various crisis periods.

**Fig 2** depicts the dynamics of agricultural futures prices. It shows that these prices have experienced cycles of boom and crash. Moreover, the price dynamics between China and the US are highly correlated. Besides, the periods are noteworthy during the GFC (August 9, 2007–April 2, 2009), the climate disasters (2010–2014), the US-China trade war (January 22, 2018–December 31, 2022), the COVID-19 pandemic (December 31, 2019–December 31, 2022), and the Russia-Ukraine war (February 24–December 31, 2022). According to the Global Report on Food Crises in 2021, conflict, extreme weather, and economic shocks are the three key driving factors of acute food insecurity. This paper focuses on extreme risk spillovers in agricultural futures markets in crises with significant economic and geopolitical shocks; the impact of extreme weather shocks is not discussed in depth in the subsequent analysis.

During the GFC, agricultural futures prices in China and the US fluctuated wildly. The prices rose rapidly and then fell after the introduction of bailout measures in various countries. In 2010–2014, the forward movement of prices might be related to market supply shocks caused by extreme climate disasters. For example, in 2011–2012, a drought in the US, France, Germany, Brazil, Argentina, and other countries led to a reduction in crop yields, thus driving price fluctuations. During the US-China trade war, agricultural futures prices in the US and China initially rose (except for sugar and soybean oil) and then oscillated at a low level. After the outbreak of COVID-19, the two countries’ markets also showed highly volatile. The COVID-19 pandemic affected all six commodities and brought a tremendous price increase. In February 2022, the Russia-Ukraine war caused a further rise in the prices of US and Chinese agricultural futures. Remarkably, most prices reached new peaks, surpassing the levels seen during the GFC.

The descriptive statistics of the log return series of agricultural commodity futures in the US and China are presented in **Table 3**. We find that the average returns of all agricultural futures are close to zero. Regarding volatility, the standard deviation of returns is generally larger in the US than in the Chinese markets, implying that the US futures markets are generally riskier. The skewness and kurtosis statistics and the Jarque-Bera (JB) normality test results suggest that the distribution of all return series exhibits more asymmetry, thicker tails, and higher peaks compared to the Gaussian distribution.

**Table 3:** Descriptive statistics for the returns.

	Soybean	Wheat	Sugar	Corn	Soybean Meal	Soybean Oil
*Panel A*: *Chinese markets*						
Mean	0.0002	0.0002	0.0001	0.0002	0.0002	0.0002
Std. Dev.	0.0118	0.0099	0.0117	0.0082	0.0143	0.0140
Skewness	0.0546	0.0779	0.1993	0.3986	−0.9249	−0.3179
Kurtosis	32.2822	51.6376	7.2665	16.2614	11.6518	6.8955
Min	−0.1655	−0.1508	−0.0773	−0.0659	−0.1641	−0.1161
Max	0.1775	0.1386	0.0991	0.0919	0.0762	0.0702
Obs	4129.0000	4129.0000	4129.0000	4129.0000	4129.0000	4129.0000
JB	147518***	406988***	3159***	30365***	13466***	2680***
ADF	−66.5296***	−64.6618***	−64.6952***	−66.2264***	−65.6583***	−65.3950***
PP	−66.5296***	−64.6618***	−64.6952***	−66.2264***	−65.6583***	−65.3950***
Q (5)	22.4432	27.2997	23.3204	34.3233**	24.9362	48.0453***
Q2 (20)	30.8719*	50.9337***	644.9905***	39.4929***	148.1482***	965.2056***
ARCH (10)	13.1996	47.9922***	217.0153***	30.6981***	48.6770***	308.7696***
*Panel B*: *US markets*						
Mean	0.0002	0.0002	0.0001	0.0003	0.0002	0.0003
Std. Dev.	0.0160	0.0226	0.0212	0.0202	0.0195	0.0161
Skewness	−1.6657	−0.0716	0.2975	−2.6461	−1.6142	−0.5638
Kurtosis	19.8648	15.2042	9.5492	49.7667	19.9551	10.6888
Min	−0.2235	−0.2289	−0.1243	−0.3986	−0.2509	−0.1874
Max	0.0740	0.2581	0.2210	0.1088	0.1094	0.0762
Obs	4129.0000	4129.0000	4129.0000	4129.0000	4129.0000	4129.0000
JB	50841***	25627***	7440***	381095***	51250***	10389***
ADF	−64.4242***	−66.3991***	−65.0505***	−65.0769***	−66.0024***	−63.7729***
PP	−64.4242***	−66.3991***	−65.0505***	−65.0769***	−66.0024***	−63.7729***
Q (5)	39.6696***	48.4027***	16.7301	25.6060	23.3707	24.7219
Q2 (20)	165.2570***	591.5012***	150.8207***	21.8149	252.9679***	886.8916***
ARCH (10)	73.8959***	431.0398***	67.8981***	19.5752**	140.7390***	333.7734***

Note: JB represents the Jarque-Bera test for normality; ADF and PP respectively denote the estimates of the Augmented-Dickey-Fuller and Phillips-Perron unit root tests; Q (5) and Q2 (20) are the Ljung-Box tests for serial correlation in returns and squared returns; ARCH(10) is the Engle’s Lagrange multiplier test for autoregressive conditional heteroskedasticity (ARCH) of order 10

***, **, and * denote significance at 1%, 5%, and 10% levels, respectively.

The results of unit-root tests suggest that all return series exhibit statistical stationarity at the 5% significance level. Furthermore, the Ljung-Box test statistics show significant serial autocorrelation in most squared return series. In addition, we find that most return series have ARCH effects. Hence, it is appropriate for us to adopt the ARMA-GARCH model to capture the characteristics of asymmetry, fat tails, and volatility persistence for the return series.

The correlations of returns in the US-China pair markets are presented in **Table 4**. It shows significant correlations for all commodity futures using all samples. These findings indicate that trading information in the daytime in one market can act as necessary “overnight” news affecting the other market. This motivates our analysis of the bidirectional risk spillovers between Chinese and US markets in the next section. Furthermore, we find their correlations are generally more substantial during the GFC. After the US-China trade war, the correlations go down. During the COVID-19 pandemic and the Russia-Ukraine war in 2022, correlations between the pair markets increase again. Therefore, it will be necessary to consider the potential changes in the dependence structure during periods of financial stress.

**Table 4:** Pearson correlations of returns in the US-China pair markets.

	Soybean	Wheat	Sugar	Corn	Soybean Meal	Soybean Oil
*Panel A: between* $R_{t}^{i,China}$ and $R_{t}^{i,US}$						
All sample	0.1732***	0.0858***	0.1136***	0.1129***	0.2075***	0.2645***
GFC	0.3530***	0.1347***	0.1399***	0.2310***	0.2432***	0.3476***
US-China trade war	0.0239	0.0051	0.0863*	0.0239	0.0956**	0.0619
COVID-19 pandemic	0.0386	0.1321***	0.0553	0.1350***	0.3198***	0.3203***
Russia-Ukraine war in 2022	0.1354*	0.2095***	0.1205*	0.2623***	0.3311***	0.3874***
*Panel B: between* $R_{t+1}^{i,China}$ and $R_{t}^{i,US}$						
All sample	0.2424***	0.1317***	0.3241***	0.1365***	0.2944***	0.3292***
GFC	0.3519***	0.1497***	0.1969***	0.1041**	0.2889***	0.3584***
US-China trade war	0.1210**	0.1897***	0.2571***	0.0251	0.1257***	0.2612***
COVID-19 pandemic	0.1763***	0.0907**	0.4783***	0.2270***	0.3017***	0.3275***
Russia-Ukraine war in 2022	0.3085***	0.0466	0.4897***	0.2263***	0.2561***	0.2293***

Note: This table only reports the correlations of the same type of commodities between the US and China

***, **, and * denote significance at 1%, 5%, and 10% levels, respectively.

## 5. Empirical results

### 5.1 Estimation of dependence-switching copula models

This section uses ARMA-GARCH models to estimate the marginal distributions of the return series. The lags of ARMA and GARCH-family models are determined by BIC. The final estimation results are shown in **Table 5**. The results show that the coefficients of *α*’ and *β*’ are generally significant, indicating heteroscedastic and long-term memory effects for the volatility in the futures markets. The estimated results of the shape parameters of US agricultural futures are larger than those of Chinese futures. It indicates that the US markets tend to have a heavier tail and a higher probability of extreme risk than the Chinese markets.

**Table 5:** Estimations of the marginal distribution models.

	Soybean	Wheat	Sugar	Corn	Soybean Meal	Soybean Oil
*Panel A*: *Chinese markets*						
*μ*	0.0001	0.0000	−0.0000	0.0002***	0.0001	−0.0000
	(0.8013)	(0.3617)	(−0.0016)	(2.6786)	(0.7129)	(−0.3454)
*ω*	0.0000	−0.1519***	−0.1171***	0.0001	0.0002***	0.0001***
	(1.4178)	(−26.8003)	(−22.6717)	(0.3681)	(4.2318)	(4.1108)
*α*’	0.0486**	0.0503***	−0.0012	0.0975	0.0624***	0.0610***
	(2.3775)	(2.7838)	(−0.1153)	(0.8629)	(14.0485)	(17.4033)
*β*’	0.9502***	0.9813***	0.9871***	0.9330***	0.9462***	0.9517***
	(58.2017)	(3795.6958)	(1723.8108)	(10.2575)	(684.4850)	(9245.0848)
*γ*		0.2562***	0.1189***			
		(7.1507)	(5.7285)			
Skewness					0.9469***	
					(50.4252)	
Shape	3.0358***	2.1000***	1.0289***	2.7462***	3.5883***	0.8196***
	(11.6322)	(156.7234)	(19.4039)	(18.0888)	(17.6322)	(19.8397)
*Panel B*: *US markets*						
*μ*	0.0004*	0.0002	−0.0002	0.0004	0.0002	0.0001
	(1.6562)	(0.9034)	(−0.5425)	(1.5624)	(0.9426)	(0.3125)
*ω*	0.0000	0.0003***	0.0002***	0.0000	0.0002*	0.0000***
	(1.2022)	(6.7580)	(4.2247)	(0.4823)	(1.9211)	(5.9815)
*α*’	0.0597***	0.0552***	0.0550***	0.0697*	0.0653***	0.0413***
	(7.0609)	(15.1745)	(17.0626)	(1.7868)	(3.1830)	(17.4162)
*β*’	0.9417***	0.9423***	0.9504***	0.9168***	0.9414***	0.9559***
	(94.8130)	(6149.1946)	(10148.1640)	(54.1047)	(47.1816)	(1297.3212)
*γ*						
					
Skewness	0.9444***	1.0909***				
	(48.1692)	(47.6935)				
Shape	4.4866***	5.4310***	1.1888***	4.0897***	4.0733***	7.5764***
	(14.3887)	(10.5944)	(26.9262)	(6.8182)	(14.6145)	(8.3816)

Note: This table reports the results of maximum likelihood estimates. *μ*_*i*_, *ω*_*i*_, *α*’_*i*,*k*_, *β*’_*i*,*k*_, and *γ* are parameters in Eqs (1) and (3), denoting the constant of the mean equation, constant of variance equation, ARCH effects, GARCH effects and asymmetric effects, respectively. The values in parenthesis are the *t* statistics

***, **, and * denote significance at 1%, 5%, and 10% levels, respectively.

Based on the standardized residuals estimated by these marginal distribution models, we further employ copula models to capture their dependence. We choose the optimal copula functions based on the log-likelihood values to describe the tail dependence, as displayed in **Table 6**. The results for copula model estimations are shown in **Table 7**. This study estimates the copula model using the IFM method, where the standard errors are computed through the Godambe matrix. The results show that most parameters exhibit significant deviations from 0. In the copula model, we categorize the regimes in Eq (6) as low and high dependence, with 0 indicating low dependence and 1 indicating high dependence. The notable disparity observed in the intercept terms between the 0 and 1 states provides evidence supporting the shift from low to high dependence.

**Table 6:** The selected optimal copula based on log-likelihood values.

Type	Best copula selection	Type	Best copula selection
*Panel A: between* $R_{t}^{i,China}$ and $R_{t}^{i,US}$		*Panel B: between* $R_{t+1}^{i,China}$ and $R_{t}^{i,US}$	
Soybean	MSTV SJC	Soybean	MSTV SJC
Wheat	MSTV Gaussian	Wheat	MSTV t
Sugar	MSTV SJC	Sugar	MSTV SJC
Corn	MSTV rotated Gumbel	Corn	MSTV SJC
Soybean Meal	MSTV SJC	Soybean Meal	MSTV t
Soybean Oil	MSTV SJC	Soybean Oil	MSTV Gaussian

**Table 7:** Estimated parameters of copula model.

*Panel A: between* $R_{t}^{i,China}$ and $R_{t}^{i,US}$								
	Soybean	Sugar	Soybean Meal	Soybean oil			Wheat	Corn
MSTV SJC copula					MSTV Gaussian /rotated Gumbel copula			
$\psi_{U,0,s_{t}=0}$	−10.0377	−9.8402***	−4.9641***	−4.3947***		$\psi_{0,s_{t}=0}$	0.0917	0.1778
	(−0.2537)	(−2.7489)	(−3.1513)	(−4.7480)			(0.9073)	(0.9921)
$\psi_{L,0,s_{t}=0}$	−9.9935	−9.2813**	−4.9951***	−2.4204		$\psi_{0,s_{t}=1}$	0.5057***	0.4497***
	(−0.1693)	(−2.0996)	(−3.1989)	(−0.7129)			(5.2687)	(3.0998)
$\psi_{U,0,s_{t}=1}$	0.4274	−6.9998	−3.5287***	−2.6470***		*ψ*_1_	−2.0018***	−0.1920**
	(0.0033)	(−1.4622)	(−3.2219)	(−4.1206)			(−379.1784)	(−2.0163)
$\psi_{L,0,s_{t}=1}$	−0.6469	−2.0200	−1.7246	1.4317		*ψ*_2_	0.1524	0.2883
	(−0.0159)	(−1.4445)	(−1.2050)	(1.0517)			(1.1916)	(0.9146)
*ψ*_*U*,1_	0.0006	3.2537*	4.9954	3.4956				
	(0.0000)	(1.6656)	(1.0825)	(0.9566)				
*ψ*_*U*,2_	0.0004	−4.6450	1.4683	−4.7916***				
	(0.0000)	(−0.2700)	(0.5915)	(−2.7792)				
*ψ*_*L*,1_	−11.1452	12.5685	2.4412	1.3946				
	(−0.0237)	(1.3226)	(0.6054)	(0.9225)				
*ψ*_*L*,2_	0.4255	0.4175	1.8547	−9.6505				
	(0.0295)	(0.3987)	(0.6858)	(−1.5812)				
*Prob*_00_	0.6598	0.6750	0.6743	0.6749		*Prob*_00_	0.6599	0.6243
	(0.0023)	(0.3998)	(0.5923)	(0.4267)			(0.5297)	(0.4643)
*Prob*_11_	0.6598	0.6750	0.6747	0.6749		*Prob*_11_	0.6599	0.6225
	(0.0026)	(0.8564)	(1.1012)	(0.5742)			(0.5176)	(0.0595)
*Panel B: between* $R_{t+1}^{i,China}$ and $R_{t}^{i,US}$								
	Soybean	Sugar	Corn		Wheat	Soybean Meal		Soybean Oil
MSTV SJC copula				MSTV t copula			MSTV Gaussian copula	
$\psi_{U,0,s_{t}=0}$	−4.5908	−2.5564***	−9.8665	$\psi_{0,s_{t}=0}$	0.4530	−0.0113**	$\psi_{0,s_{t}=0}$	0.2957
	(−0.2051)	(−4.6192)	(−0.3100)		(0.2079)	(−1.9755)		(1.5765)
$\psi_{L,0,s_{t}=0}$	−3.0923	−2.8777**	−7.4342	$\psi_{0,s_{t}=1}$	1.2186*	0.2015***	$\psi_{0,s_{t}=1}$	1.2034*
	(−0.1022)	(−2.4578)	(−0.6941)		(1.7936)	(5.2553)		(1.9423)
$\psi_{U,0,s_{t}=1}$	−2.7618	−1.9774***	−5.6588	*ψ*_1_	−2.0243***	1.9706***	*ψ*_1_	0.2610
	(−0.6534)	(−13.7448)	(−0.1503)		(−10.0436)	(67.9961)		(0.2630)
$\psi_{L,0,s_{t}=1}$	−0.7970	−0.4228	0.5571	*ψ*_2_	−0.1297	0.0326**	*ψ*_2_	−0.2522*
	(−0.0555)	(−0.7463)	(0.0464)		(−0.2183)	(2.2654)		(−1.7082)
*ψ*_*U*,1_	4.8671	4.2690***	−9.6818					
	(0.0472)	(16.1223)	(−0.2155)					
*ψ*_*U*,2_	−4.9864	−4.8210***	−1.1538					
	(−0.1040)	(−5.0831)	(−0.0689)					
*ψ*_*L*,1_	0.9236	−0.7161	13.1413	$\vartheta_{s_{t}=0}$	1.6558	7.3104***		
	(0.0206)	(−1.5921)	(0.1546)		(0.1124)	(6.0895)		
*ψ*_*L*,2_	2.1478	2.4645	−9.3029	$\vartheta_{s_{t}=1}$	−1.4420**	0.3721		
	(0.0421)	(0.7563)	(−0.1695)		(−2.0476)	(0.5210)		
*Prob*_00_	0.6747	0.6747	0.6749	*Prob*_00_	0.6746	0.6653***	*Prob*_00_	0.6729
	(0.0316)	(0.8237)	(0.0416)		(0.0344)	(2.9524)		(1.6409)
*Prob*_11_	0.6747	0.6747	0.6749	*Prob*_11_	0.6747	0.6630***	*Prob*_11_	0.6726*
	(0.0176)	(1.0908)	(0.0679)		(0.0280)	(2.7111)		(1.7251)

Note: *ψ*_0_, *ψ*_1_ and *ψ*_2_ represent dependence, persistence, and adjustment, respectively. The subscripts *s*_*t*_ = 0 and *s*_*t*_ = 1 assume the low and high dependence, respectively. The subscripts *U* and *L* denote the upside and downside tail, respectively. The values in parenthesis are *t* statistics

***, **, and * denote significance at the 1%, 5%, and 10% levels, respectively.

**Fig 3:**
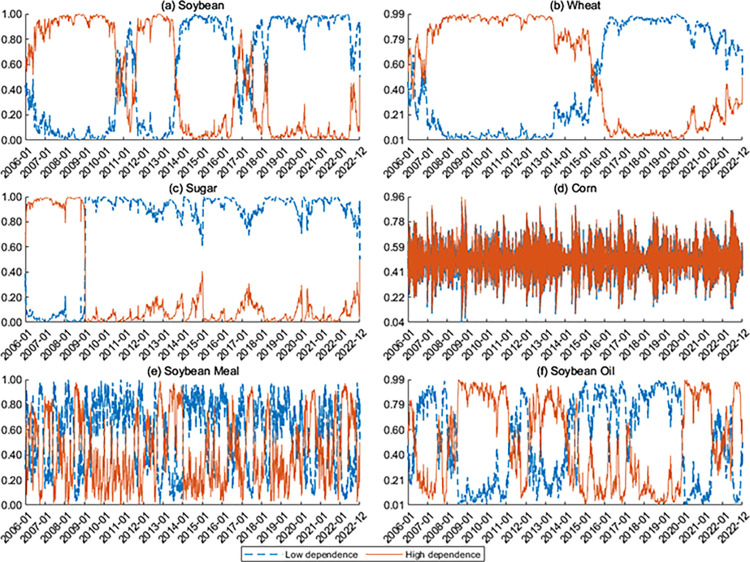
Smoothed probabilities for the regimes of dependence between $R_{t}^{i,China}$ and $R_{t}^{i,US}$.

**Fig 4:**
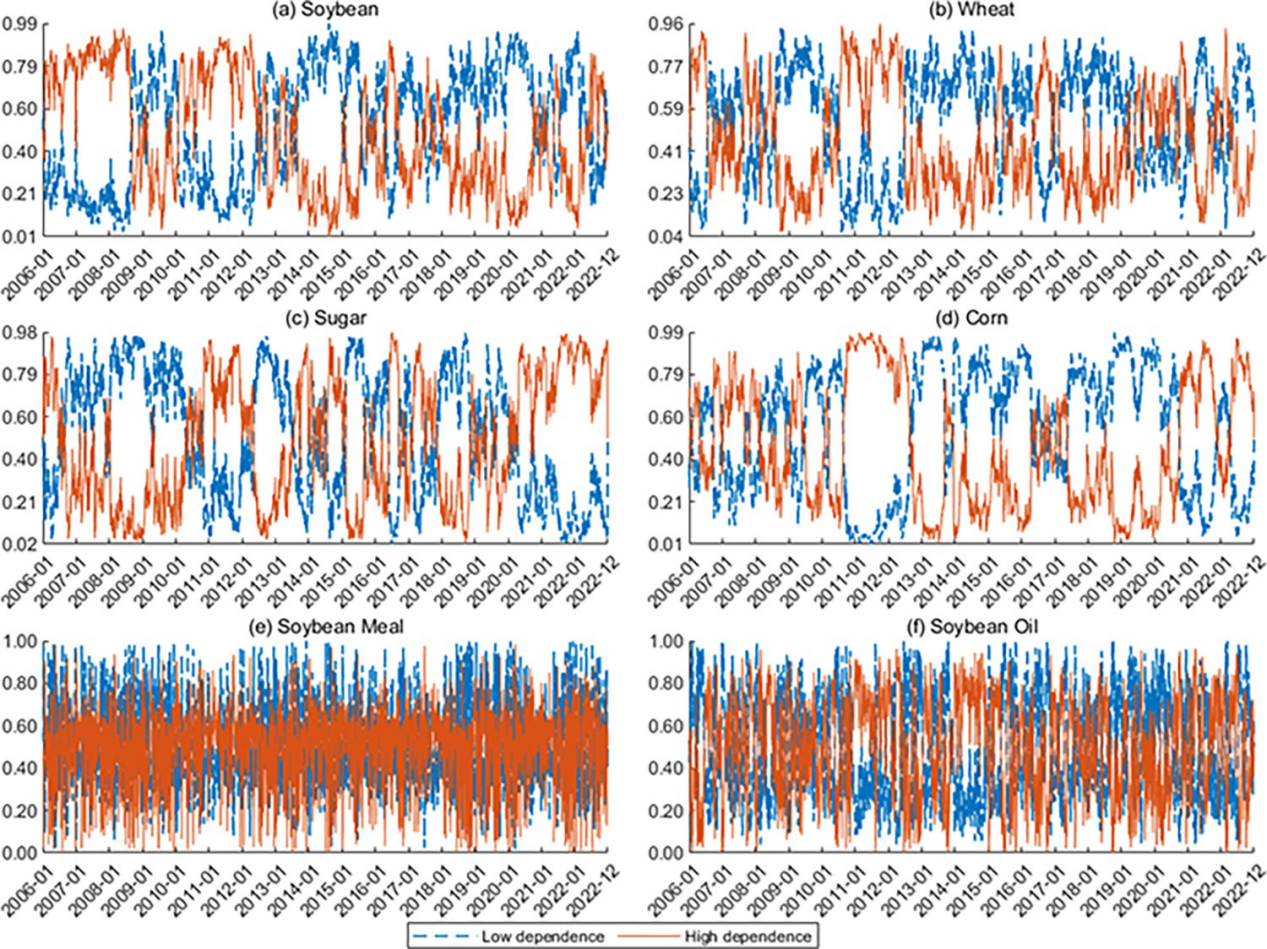
Smoothed probabilities for the regimes of dependence between $R_{t+1}^{i,China}$ and $R_{t}^{i,US}$.

The smoothed probabilities for the regimes of low and high dependence are shown in **Figs 3** and **4**. The sum of the probabilities associated with low and high dependence is always one at any given time. For the dependence between $R_{t}^{i,China}$ and $R_{t}^{i,US}$, the smoothing probabilities for soybean futures can be roughly divided into two intervals: higher dependence before 2014 and lower ones after 2014. The dependence is in the transition period in 2010–2012 and 2016–2018. For wheat, dependence is high before 2015 and low after 2015. For sugar, the dependence between the US and Chinese markets is high before 2009 and low after 2009. Notably, the smoothing probability of high dependence on soybean, wheat, and sugar gradually increases in 2022. This may be related to the co-movements brought about by the events of the Russia-Ukraine conflict. Furthermore, the smoothing probability for corn, soybean meal, and soybean oil shifts frequently between low and high dependence, with high dependence remaining dominant after the Russia-Ukraine conflict in 2022.

For the dependence between $R_{t+1}^{i,China}$ and $R_{t}^{i,US}$, the dependence for soybean futures tends to be low after 2014. For sugar and corn, the high dependence remains dominant after 2020. In addition, the smoothing probabilities shift frequently between high and low dependence for wheat, soybean oil, and soybean meal. Our findings confirm that the dependence structure shows two distinct regimes for all commodity futures.

**Fig 5:**
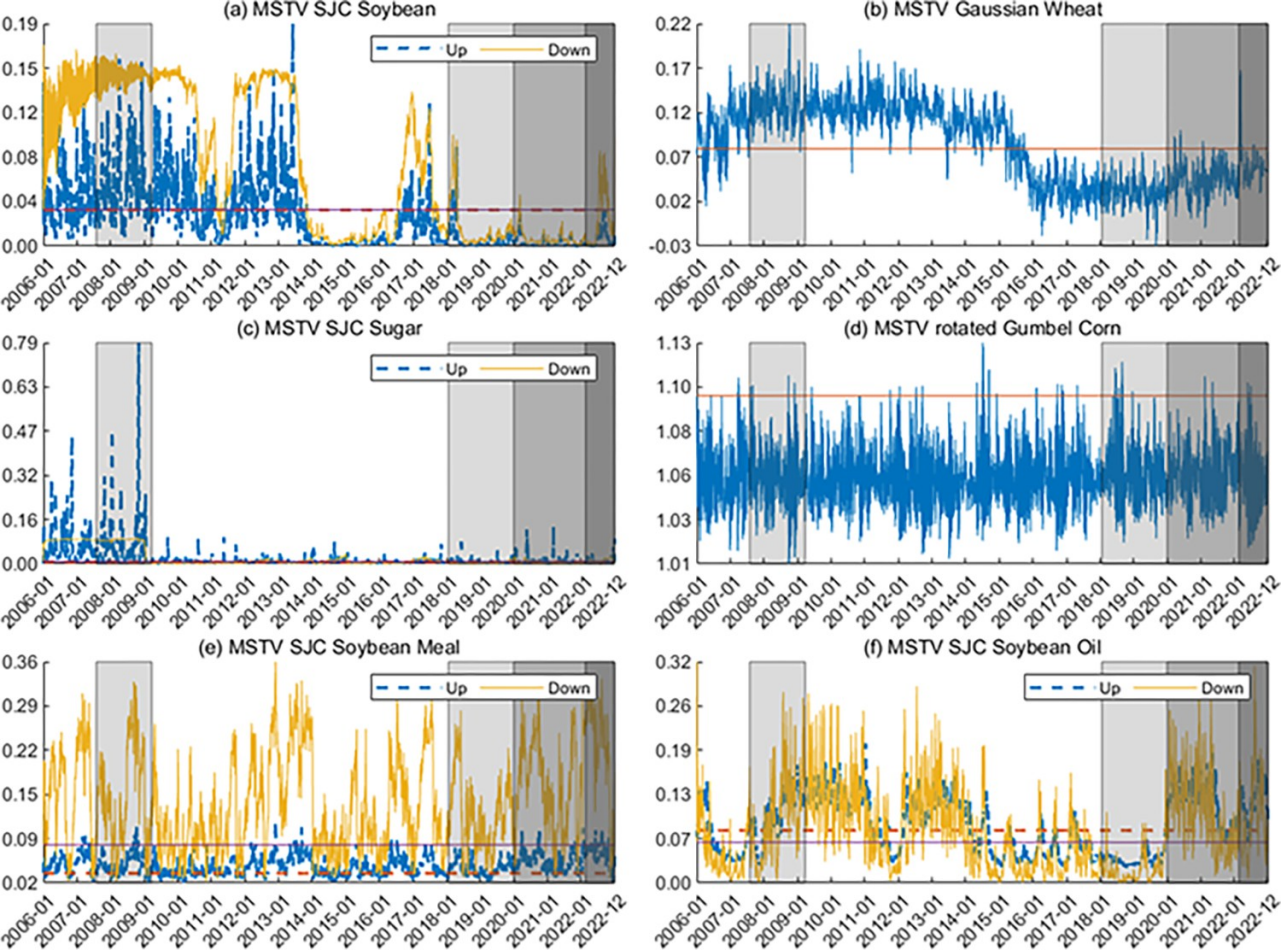
Dynamics of dependence between $R_{t}^{i,China}$ and $R_{t}^{i,US}$. The horizontal lines in the figure are static dependence parameters. For the MSTV SJC copula model, the red line indicates the upward tail dependence, and the purple line indicates the downward tail dependence. In the figure, the periods for the GFC, the US-China trade war, the COVID-19 pandemic, and the Russia-Ukraine war are shaded.

**Fig 6:**
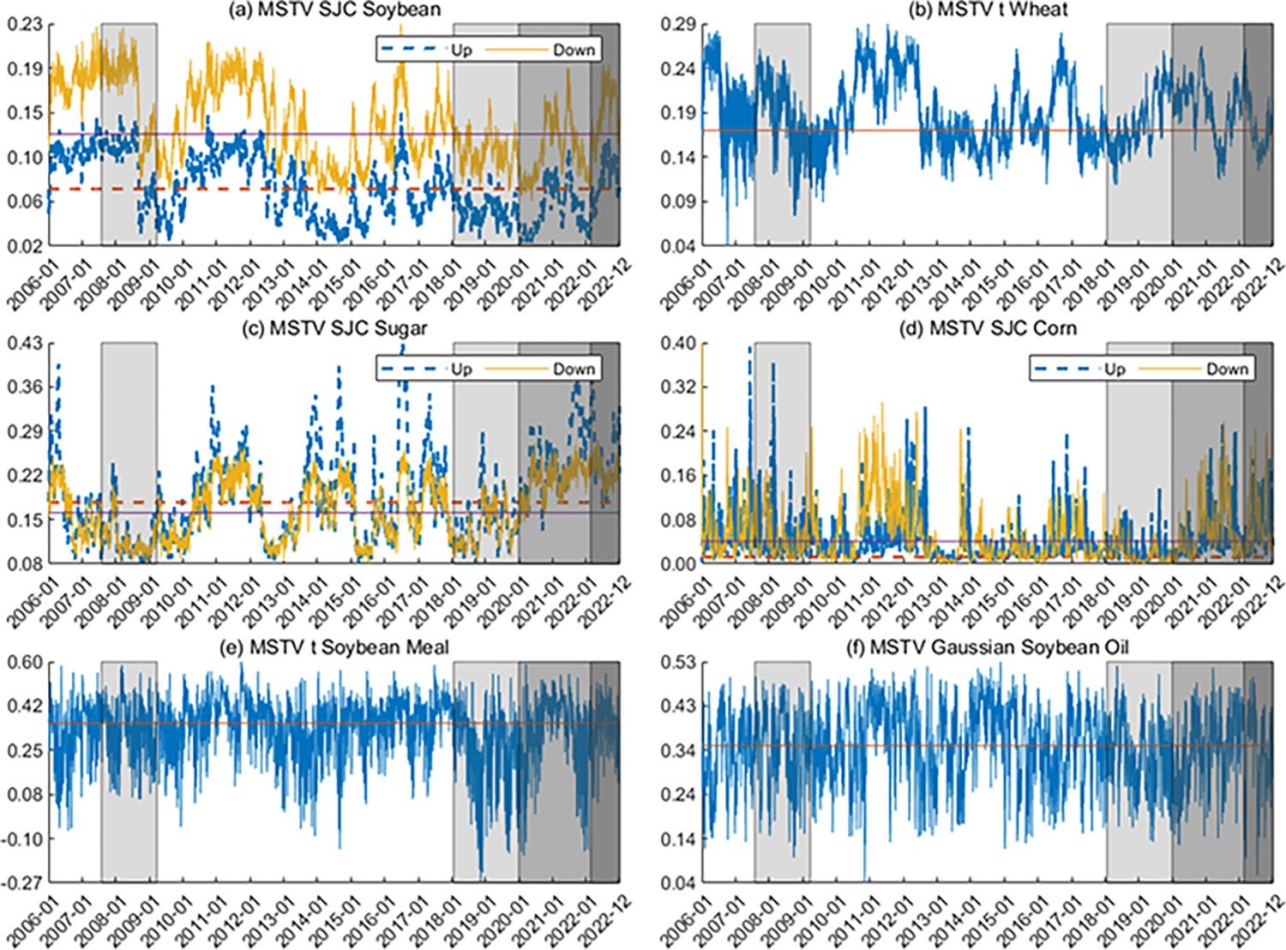
Dynamics of dependence between $R_{t+1}^{i,China}$ and $R_{t}^{i,US}$. The horizontal lines in the figure are static dependence parameters. For the MSTV SJC copula model, the red line indicates the upward tail dependence, and the purple line indicates the downward tail dependence. In the figure, the periods for the GFC, the US-China trade war, the COVID-19 pandemic, and the Russia-Ukraine war are shaded.

**Figs 5 and 6** show the time-varying tail dependence between the US and Chinese markets. The dependence between agricultural futures in the two markets is almost positive, indicating that they tend to move in the same direction. Furthermore, the tail dependence parameters exhibit lower values in the low dependence state and higher values in the high dependence state, in line with the results for smoothing probabilities. In addition, we find that the dependence between China and the US generally shows high dependence during the GFC, the COVID-19 pandemic, and the Russia-Ukraine war in 2022. This finding is consistent with the original intention of the copula model with Markov switching regimes.

### 5.2 Analysis of extreme risk spillovers

First, this section discusses the significance of extreme risk spillover effects, as shown in **Table 8**. Regarding downside risk spillovers, except for corn, CoVaR values for both markets are smaller than the benchmark CoVaR (*CoVaR50*), confirming the existence of downside risk spillovers between the US and Chinese markets. As for the upside risk spillovers, the CoVaR values are larger than the benchmark CoVaR in both markets for all six commodities. Furthermore, based on the Kolmogorov-Smirnov (K-S) test statistics, we find that ΔCoVaR values are significantly unequal to zero. It confirms the significance of the upside/downside extreme risk spillovers in Chinese and US pair markets for all six commodities (except for corn). This finding highlights the importance of managing extreme risk spillovers between these markets. Neglecting to address these risk spillovers in cross-country markets may lead to underestimating fluctuations in these markets.

**Table 8:** Summarization of the VaR, CoVaR, and ΔCoVaR for agricultural futures.

	Downside				Upside			
	*VaR*	*CoVaR50*	*CoVaR5*	*ΔCoVaR*	*VaR*	*CoVaR50*	*CoVaR95*	*ΔCoVaR*
*Panel A*: *VaR in Chinese markets and risk spillovers from the US to China*								
Soybean	−2.7858	−3.3166	−8.2063	−4.8896***	2.8038	−3.3351	2.3609	5.6960***
	(1.0308)	(1.2028)	(2.9856)	(1.7831)	(1.0308)	(2.7560)	(0.8511)	(3.4016)
Wheat	−5.1111	−5.9361	−19.5669	−13.6308***	5.1160	−40.7045	3.8438	44.5484***
	(1.6801)	(1.9769)	(6.5224)	(4.5455)	(1.6801)	(33.1296)	(1.2817)	(33.6756)
Sugar	0.0479	0.0300	0.0023	−0.0277***	3.4251	0.0006	3.0852	3.0846***
	(0.0142)	(0.0093)	(0.0007)	(0.0086)	(1.0159)	(0.0004)	(0.9054)	(0.9052)
Corn	−2.1504	−2.3955	−6.4354	−4.0399***	2.1890	−11.2407	1.7219	12.9626***
	(0.7517)	(0.8489)	(2.2694)	(1.4206)	(0.7517)	(3.9754)	(0.6011)	(4.5336)
Soybean Meal	−1.2654	−1.2929	−1.3805	−0.0876***	2.4776	−1.4485	2.0772	3.5257***
	(0.3419)	(0.3500)	(0.3730)	(0.0237)	(0.6589)	(0.4164)	(0.5883)	(0.9748)
Soybean Oil	0.2074	0.1553	0.0292	−0.1262***	3.8289	0.0105	3.5663	3.5558***
	(0.0761)	(0.0599)	(0.0116)	(0.0482)	(1.3953)	(0.0061)	(1.2885)	(1.2841)
*Panel B*: *VaR in US markets and risk spillovers from China to the US*								
Soybean	−1.4382	−1.4631	−1.5741	−0.1111***	2.7679	−1.5389	2.1181	3.6570***
	(0.4596)	(0.4706)	(0.5037)	(0.0336)	(0.8518)	(0.5218)	(0.6865)	(1.2008)
Wheat	−2.1473	−2.1344	−2.2914	−0.1570***	4.0140	−2.3060	2.6295	4.9354***
	(0.5907)	(0.5888)	(0.6302)	(0.0446)	(1.0853)	(0.6333)	(0.7351)	(1.3583)
Sugar	0.0114	0.0038	−0.0156	−0.0193***	6.5629	−0.0162	5.1296	5.1457***
	(0.0074)	(0.0056)	(0.0002)	(0.0054)	(1.7621)	(0.0001)	(1.4063)	(1.4063)
Corn	−4.0158	4.2942	9.0566	4.7624***	4.0868	−0.7992	0.7102	1.5094***
	(1.6154)	(1.6968)	(3.5939)	(1.8971)	(1.6154)	(0.3402)	(0.2668)	(0.6031)
Soybean Meal	−3.8227	−4.1627	−8.8594	−4.6967***	3.8638	−3.3068	3.2045	6.5113***
	(1.2585)	(1.3686)	(2.9076)	(1.5391)	(1.2585)	(1.2888)	(1.0391)	(2.3081)
Soybean Oil	−2.8216	−3.1379	−5.5902	−2.4523***	2.8348	−5.5081	2.4560	7.9641***
	(0.9703)	(1.1253)	(1.9738)	(0.8491)	(0.9703)	(3.5644)	(0.8625)	(4.3723)

Note: This table presents the average and the standard deviations (in parenthesis). The values in the table have been multiplied by a factor of 100. *CoVaR50* denotes the CoVaR conditional on a regular market. *CoVaR5* and *CoVaR95* denote the CoVaR conditional on a distressed market with 5% and 95% quantile levels, respectively. The significance test of *ΔCoVaR* is performed based on the Kolmogorov-Smirnov (K-S) test. It is a non-parametric statistical test used to determine whether a sample of data follows a specific distribution. The hypothesis test is $\left\{ \begin{array}{l} H_{0}:CoVaR_{\beta ,t}^{i|j}=CoVaR_{50,t}^{i|j}\\ H_{1}:CoVaR_{\beta ,t}^{i|j}\neq CoVaR_{50,t}^{i|j} \end{array}\right.$ ***, **, and * indicate that the K-S test is significant at the 1%, 5%, and 10% levels, respectively.

**Fig 7:**
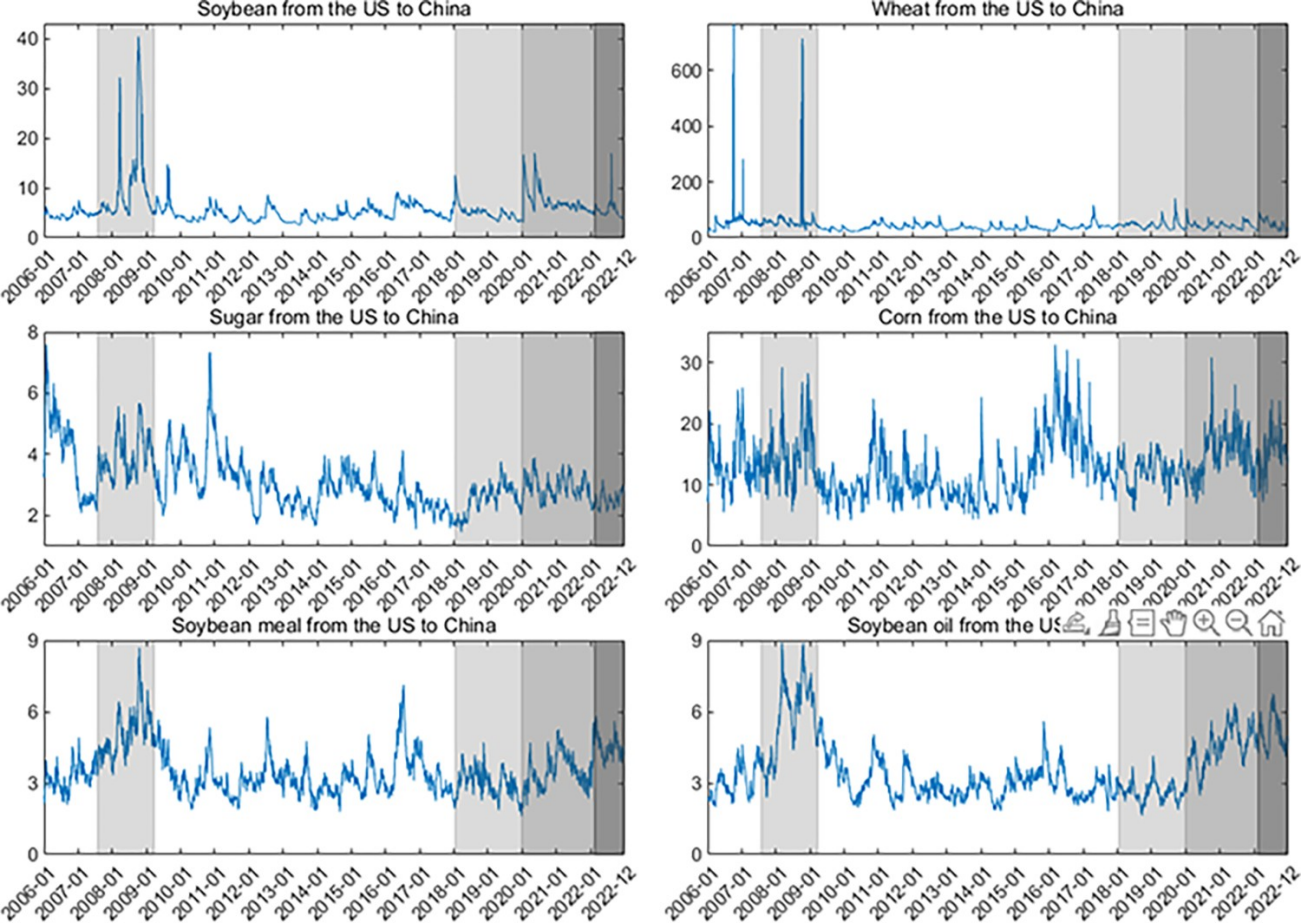
Up ΔCoVaR from the US to Chinese agricultural futures markets. All values of ΔCoVaR have been multiplied by a factor of 100.

**Fig 8:**
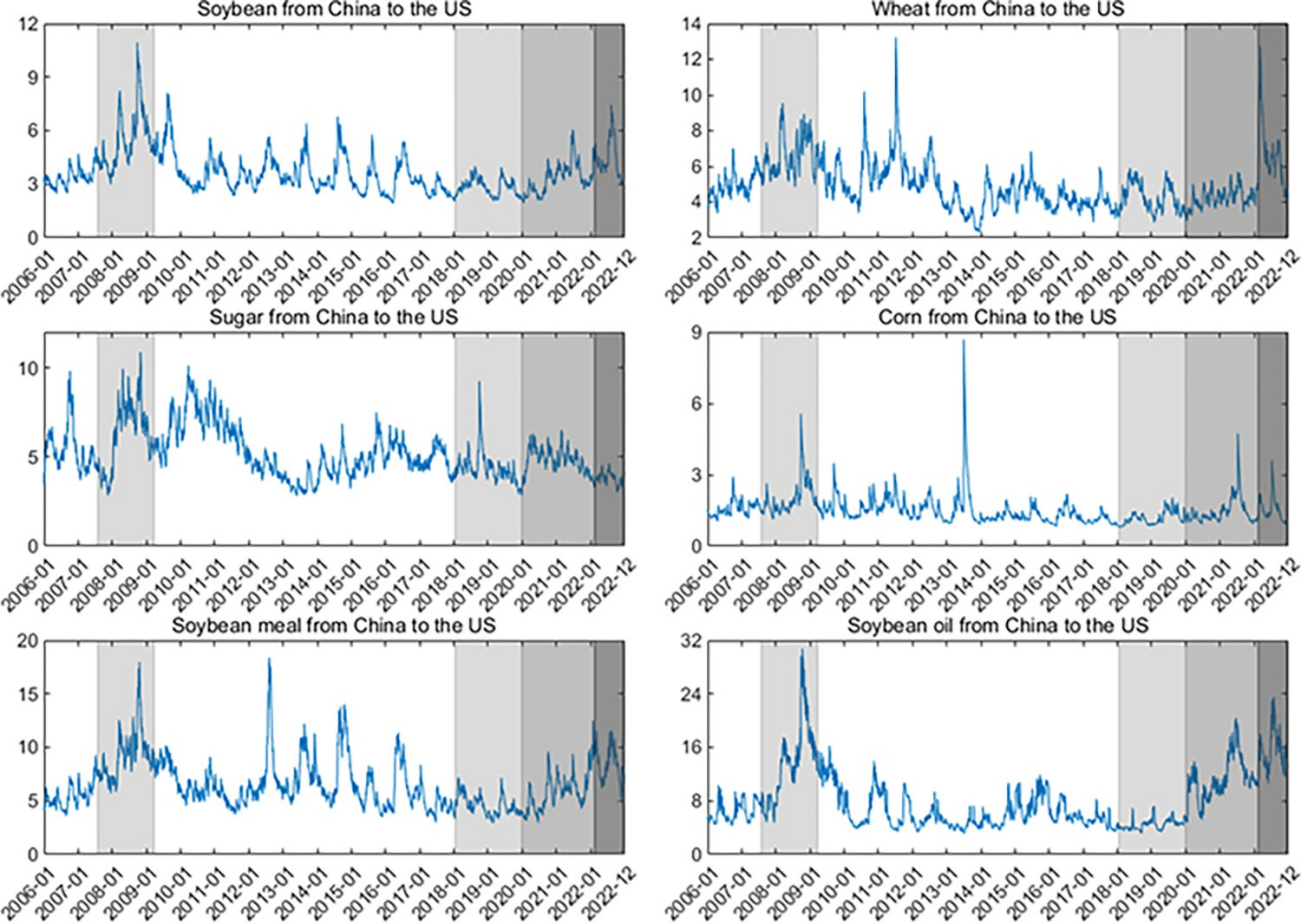
Up ΔCoVaR from the Chinese to US agricultural futures markets. All values of ΔCoVaR have been multiplied by a factor of 100.

**Fig 9:**
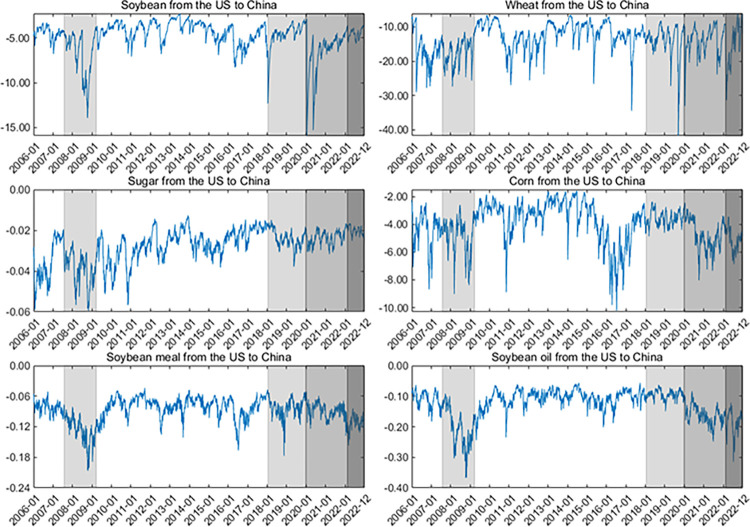
Down ΔCoVaR from the US to Chinese agricultural futures markets. All values of ΔCoVaR have been multiplied by a factor of 100.

**Fig 10:**
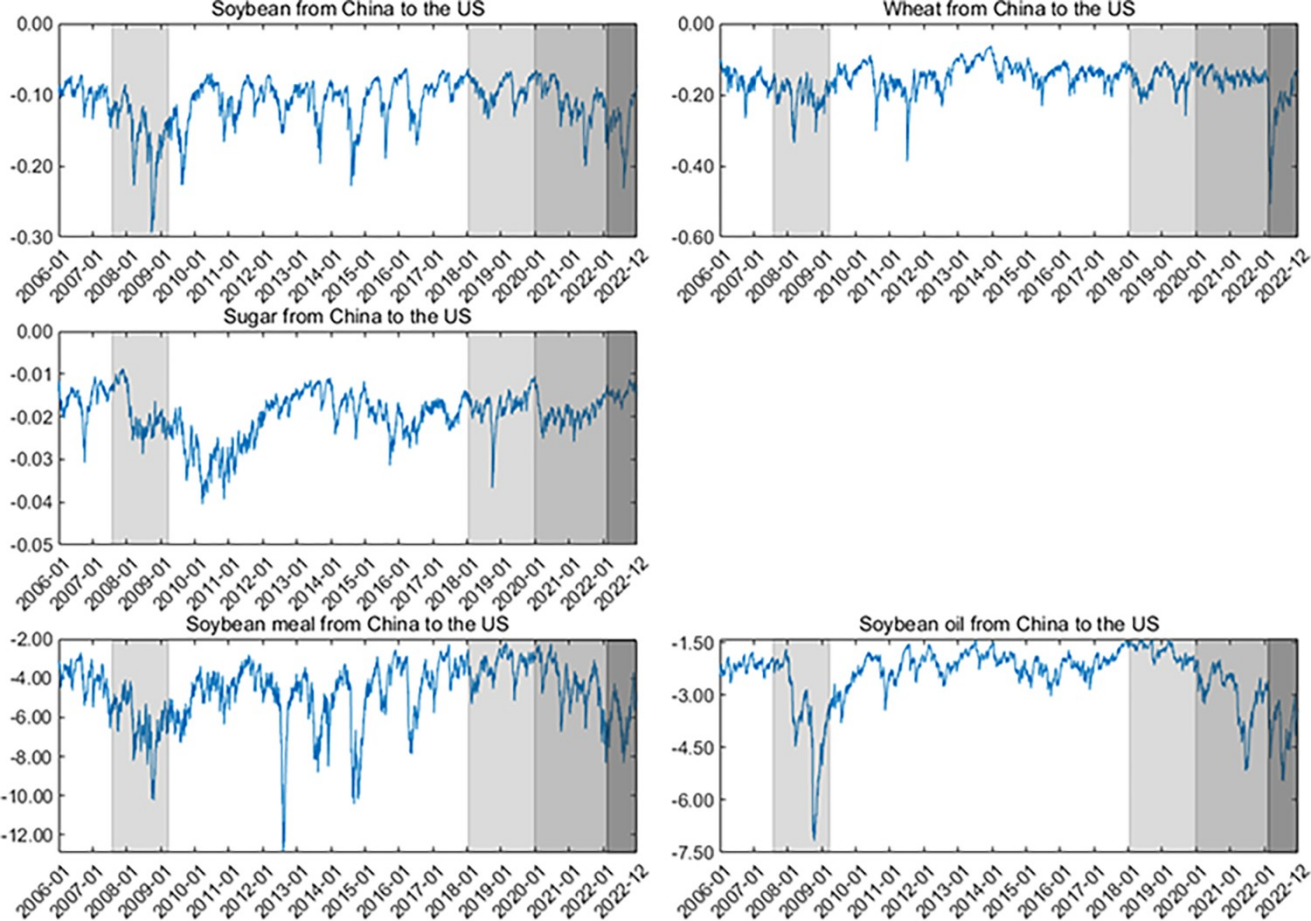
Down ΔCoVaR from the Chinese to US agricultural futures markets. All values of ΔCoVaR have been multiplied by a factor of 100. In the figure, we remove the dynamics of the risk spillovers for corn futures because their risk spillovers are not statistically significant.

Second, the dynamics of upside/downside extreme risk spillovers (measured by ΔCoVaR) are shown in **Figs 7–10**. Generally, the ΔCoVaRs are highly volatile during crises, and the spillover dynamics differ among these commodities. For example, the upside and downside extreme risk spillovers between the two markets generally grow during the GFC. Significantly, the upside risk spillovers from the US to China in soybean and wheat futures substantially increase even though China adopted agricultural price stabilization policies in 2008. During the US-China trade war, the risk spillovers between the two countries tend to increase first and then be low, especially for soybean and sugar. China was a major importer of US soybeans, and Chinese soybean consumption was highly dependent on the US markets before 2018. Therefore, the increase in risk spillover effects from the US on the Chinese soybean is the most evident after the outbreak of the US-China trade war.

Following the COVID-19 pandemic, the extreme risk spillovers between the US and Chinese markets increase sharply. Primarily, the downside risk spillovers for soybean futures from the US to China increase significantly due to China’s high dependence on international markets. In February 2022, the outbreak of the Russia-Ukraine war also aggravates the extreme risk spillovers between these two markets, especially for wheat futures. Disruptions in agricultural production and transportation in Russia and Ukraine (they contribute 19% of the world’s wheat supply) have led to dramatic volatility in global agricultural futures markets, increasing the magnitude of extreme risk spillover effects for wheat futures. On March 21, 2022, Argentina, the world’s leading exporter of soybean meal and soybean oil, announced that it would raise export taxes on soybean meal and soybean oil to control inflation due to the Russia-Ukraine war. This drives the increase of extreme risk spillovers for soybean oil and soybean meal futures. These findings align with Lien et al. [11] and Just and Echaust [12], who also highlight the increase in risk spillovers during turbulent times.

Third, we explore the asymmetry from directional and upside/downside aspects, as shown in **Tables 9 and 10**. Note that the downside extreme risk spillovers from China to the US are insignificant for corn, so there is no need to include them in comparison. **Table 9** shows that, for all commodities, upside risk spillovers in the two countries’ markets are more prominent than downside risk spillovers. These results suggest that US-Chinese agricultural futures markets tend to boom rather than crash jointly. It differs from the stock markets, which tend to crash together [74]. Low prices could lead to losses for long-position investors, while extremely high agricultural commodity prices could cause losses for short-position investors and jeopardize food security [12]. Thus, upward co-movements in US-Chinese markets may be of more significant concern than downward, highlighting the importance of preventing food insecurity caused by cross-border risk spillovers.

**Table 9:** K-S test for the asymmetry of upside and downside risk spillovers.

	$H_{0}:\Delta CoVaR_{5\%,t}^{i|j}=\Delta CoVaR_{95\%,t}^{i|j}$ $H_{1}:\Delta CoVaR_{5\%,t}^{i|j}>\Delta CoVaR_{95\%,t}^{i|j}$		$H_{0}:\Delta CoVaR_{5\%,t}^{i|j}=\Delta CoVaR_{95\%,t}^{i|j}$ $H_{1}:\Delta CoVaR_{5\%,t}^{i|j}<\Delta CoVaR_{95\%,t}^{i|j}$	
	The US → China	China → The US	The US → China	China → The US
Soybean	1.0000***	1.0000***	0.0000	0.0000
Wheat	1.0000***	1.0000***	0.0000	0.0000
Sugar	1.0000***	1.0000***	0.0000	0.0000
Corn	1.0000***		0.0000	
Soybean meal	1.0000***	1.0000***	0.0000	0.0000
Soybean oil	1.0000***	1.0000***	0.0000	0.0000

Note: This table presents the K-S test statistics

***, **, and * significant at the 1%, 5%, and 10% levels, respectively.

**Table 10:** K-S test for the asymmetry of risk spillovers from the US to China and from China to the US.

	$H_{0}:\Delta CoVaR_{\beta ,t}^{CHN|USA}=\Delta CoVaR_{\beta ,t}^{USA|CHN}$ $H_{1}:\Delta CoVaR_{\beta ,t}^{CHN|USA}>\Delta CoVaR_{\beta ,t}^{USA|CHN}$		$H_{0}:\Delta CoVaR_{\beta ,t}^{CHN|USA}=\Delta CoVaR_{\beta ,t}^{USA|CHN}$ $H_{1}:\Delta CoVaR_{\beta ,t}^{CHN|USA}<\Delta CoVaR_{\beta ,t}^{USA|CHN}$	
	Downside	Upside	Downside	Upside
Soybean	1.0000***	0.0000	0.0000	0.4886***
Wheat	1.0000***	0.0000	0.0000	1.0000***
Sugar	0.4755***	0.7013***	0.0000	0.0000
Corn		0.0000		0.9942***
Soybean meal	0.0000	0.6650***	1.0000***	0.0000
Soybean oil	0.0000	0.6187***	1.0000***	0.0000

Note: This table presents the K-S test statistics

***, **, and * significant at the 1%, 5%, and 10% levels, respectively.

From **Table 10**, the results show that upside risk spillover effects for soybean, wheat, and corn futures are more prominent from the US to China than from China to the US. Also, the downside risk spillovers for soybean meal and soybean oil are more significant from the US to China than the opposite. Generally, the US always plays a leading role in main grain futures markets, consistent with Chen and Weng [31]. Furthermore, China also plays an increasingly vital role in cross-country risk spillovers, such as the upside risk spillover for sugar, soybean meal and soybean oil futures. Overall, the risk spillover effects in the two markets are bidirectional, asymmetric, highly volatile, and susceptible to extreme crises.

### 5.3 Driving factors of extreme risk spillovers in crises

This section conducts a panel data regression analysis to explore which factors drive the extreme risk spillovers in crises. First, we perform monthly averaging of the daily extreme risk spillover to ensure that its data frequency is consistent with the mechanism variables. Thus, we obtain 204 monthly observations for each commodity from January 2006 to December 2022. **Table 11** summarizes all variables in the regression analysis. The statistical measures provided include sample size, mean, standard deviation, as well as minimum value, percentiles (25th, 50th, and 75th), and maximum value for each variable.

**Table 11:** Descriptive statistics of the sample from January 2006 to May 2020.

Variable	N	Mean	Std. Dev.	Min	P25	P50	P75	Max	Fisher-type unit-root test
$\Delta CoVaR_{95\%,t}^{CHN|USA}$	1224	12.2611	17.7218	1.7499	3.1123	4.6131	12.5930	264.6062	265.9324***
$\Delta CoVaR_{5\%,t}^{CHN|USA}$	1224	−3.8057	5.1974	−29.4208	−5.2433	−1.0873	−0.0797	−0.0138	234.2057***
$\Delta CoVaR_{95\%,t}^{USA|CHN}$	1224	4.9471	2.9725	0.8287	3.2559	4.5422	5.9501	26.8439	205.3772***
$\Delta CoVaR_{5\%,t}^{USA|CHN}$	1224	−0.4459	3.0273	−11.3369	−2.1948	−0.1302	−0.0186	13.9188	224.0129***
*VIX*_*t*_	1224	1.9858	0.8778	1.0125	1.3971	1.7519	2.3240	6.2639	206.9133***
*GEPU*_*t*_	1224	161.9055	75.9374	48.9513	105.1235	143.5834	204.6024	428.1037	84.9449***
*EXR*_*t*_	1224	6.7345	0.4825	6.0509	6.3613	6.6971	6.8958	8.0654	79.3575***
*GPR*_*t*_	1224	95.5181	27.3422	60.6016	80.5665	89.9050	103.1089	324.2259	243.9859***
*GDP*_*USA*,*t*_	1224	0.9988	0.0132	0.9168	0.9968	0.9995	1.0045	1.0185	109.0731***
*GDP*_*CHN*,*t*_	1224	1.0008	0.0132	0.8606	0.9955	1.0023	1.0066	1.0195	432.5238***
*TPU*_*USA*,*t*_	1224	138.6270	238.8726	7.6726	28.2103	51.2160	101.8819	1946.6830	236.7946***
*TPU*_*CHN*,*t*_	1224	192.4905	233.7867	0.0000	44.0950	105.7950	250.3761	1425.1602	260.5038***
*Flow*_*USA→CHN*,*t*_	1224	0.0106	0.4265	−2.2363	−0.1726	0.0232	0.1756	2.1523	432.5238***
*Flow*_*CHN→USA*,*t*_	1224	0.0122	0.3340	−1.1832	−0.1637	0.0267	0.2069	1.1763	432.5238***
*Pduc*_*USA*,*t*_	1224	4.2672	1.5167	2.1353	2.8519	4.0450	5.8450	6.5546	68.4347 ***
*Pduc*_*CHN*,*t*_	1224	4.0537	1.5825	1.5129	2.7685	3.4812	5.3260	7.1890	49.5581***
*Biofuel*_*t*_	1224	0.0134	0.0035	0.0075	0.0107	0.0123	0.0163	0.0215	73.1091***

Note

***, **, and * denotes significance at the 1%, 5%, and 10% levels, respectively. All values of ΔCoVaR have been multiplied by a factor of 100.

**Table 12** shows the Pearson correlations between these variables. It shows significant correlations between the extreme risk spillovers and macro factors, such as VIX and exchange rates. Moreover, TPU, crop production, and biofuel prices are also somewhat related to the risk spillover effects. In addition, the dependent variables (except for the same variables but for different counties) have low correlations. This suggests that these variables are relatively independent and may have unique impacts on the agricultural commodity markets. This also helps to avoid the issue of multicollinearity in regression analysis.

**Table 12:** Pearson correlations of regression variables.

Variables	(1)	(2)	(3)	(4)	(5)	(6)	(7)	(8)	(9)	(10)	(11)	(12)	(13)	(14)	(15)	(16)	(17)
(1) $\Delta CoVaR_{95\%,t}^{CHN|USA}$	1.000																
(2) $\Delta CoVaR_{5\%,t}^{CHN|USA}$	−0.872*	1.000															
(3) $\Delta CoVaR_{95\%,t}^{USA|CHN}$	−0.060*	0.189*	1.000														
(4) $\Delta CoVaR_{5\%,t}^{USA|CHN}$	0.186*	−0.277*	−0.679*	1.000													
(5)*VIX*_*t*_	0.082*	−0.041	0.297*	−0.024	1.000												
(6)*GEPU*_*t*_	0.012	−0.029	−0.008	−0.012	0.289*	1.000											
(7)*EXR*_*t*_	0.069*	−0.063*	0.020	0.016	0.018	−0.158*	1.000										
(8)*GPR*_*t*_	0.006	−0.010	0.024	−0.018	−0.101*	0.114*	−0.001	1.000									
(9) *GDP*_*USA*,*t*_	0.028	−0.017	−0.048	0.006	−0.455*	−0.488*	0.179*	0.193*	1.000								
(10)*GDP*_*CHN*,*t*_	−0.011	0.003	0.018	−0.001	−0.093*	−0.136*	−0.060*	−0.017	0.029	1.000							
(11)*TPU*_*USA*,*t*_	0.008	−0.026	−0.141*	0.020	−0.109*	0.438*	0.088*	0.018	0.038	−0.395*	1.000						
(12)*TPU*_*CHN*,*t*_	−0.008	−0.007	−0.109*	0.005	−0.036	0.670*	−0.008	0.009	−0.179*	−0.153*	0.596*	1.000					
(13)*Flow*_*USA→CHN*,*t*_	0.012	0.004	0.000	0.001	−0.015	−0.009	0.035	0.045	−0.023	−0.009	0.005	−0.041	1.000				
(14)*Flow*_*CHN→USA*,*t*_	0.010	−0.002	−0.012	−0.001	−0.018	0.017	0.026	0.039	−0.026	0.014	0.039	0.013	0.377*	1.000			
(15)*Pduc*_*USA*,*t*_	−0.272*	0.252*	−0.449*	0.548*	−0.002	0.038	0.003	0.013	−0.001	−0.009	0.027	0.034	−0.001	−0.001	1.000		
(16)*Pduc*_*CHN*,*t*_	−0.119*	0.223*	−0.269*	0.398*	0.003	0.073*	−0.051	0.014	−0.030	−0.009	0.036	0.054	−0.004	−0.003	0.848*	1.000	
(17)*Biofuel*_*t*_	−0.023	0.006	0.137*	−0.018	0.105*	0.119*	−0.526*	0.081*	−0.035	0.241*	−0.246*	−0.143*	−0.076*	−0.056	−0.010	0.020	1.000

Note

* shows significance at *p*<0.05.

Next, we conduct the regression analysis. Considering the between-group heteroskedasticity, the contemporaneous correlation between groups, and the within-group autocorrelation, we use the Feasible Generalized Least Squares (FGLS) regression method to estimate our models. We also include the individual fixed effects and a time trend term in the long-run panel data model. The regression has no missing values, indicating a balanced panel with 204 months and six commodities. The regression results using all total samples are shown in **Table 13**. Columns (1)–(2) denote the risk spillovers from the US to China, and columns (3)–(4) denote the opposite direction. Columns (1) and (3) indicate the upside risk spillovers; columns (2) and (4) indicate the downside side.

**Table 13:** The regression results for all samples.

	(1)	(2)	(3)	(4)
	$\Delta CoVaR_{95\%,t}^{CHN|USA}$	$\Delta CoVaR_{5\%,t}^{CHN|USA}$	$\Delta CoVaR_{95\%,t}^{USA|CHN}$	$\Delta CoVaR_{5\%,t}^{USA|CHN}$
*VIX*_*t*_	0.0596***	−0.0040	0.0154**	−0.0018**
	(2.96)	(−1.05)	(2.26)	(−1.82)
*GEPU*_*t*_	−0.0006	0.0003	0.0012	−0.0001
	(−0.17)	(0.46)	(0.98)	(−0.40)
*EXR*_*t*_	0.3596	−0.0660	−0.0614	−0.0084
	(0.55)	(−0.38)	(−0.26)	(−0.20)
*GDP*_*USA*,*t*_	2.0195	1.5328	−1.9246	0.2301
	(0.11)	(0.39)	(−0.31)	(0.24)
*GDP*_*CHN*,*t*_	−9.7940	2.8051*	−2.6230	0.3568
	(−1.20)	(1.86)	(−0.99)	(0.93)
*GPR*_*t*_	0.0004	−0.0005	0.0017	−0.0001
	(0.09)	(−0.62)	(1.17)	(−0.63)
*TPU*_*USA*,*t*_	0.0005	−0.0002**		
	(0.87)	(−2.17)		
*TPU*_*CHN*,*t*_			−0.0001	0.0000
			(−0.49)	(0.31)
*Flow*_*USA→CHN*,*t*_	0.0188	−0.0236		
	(0.12)	(−0.92)		
*Flow*_*CHN→USA*,*t*_			0.0215	0.0003
			(0.35)	(0.03)
*Pduc*_*USA*,*t*_	0.7138	−0.0211		
	(0.69)	(−0.23)		
*Pduc*_*CHN*,*t*_			0.5453**	0.0103
			(2.32)	(0.49)
*Biofuel*_*t*_	0.7523	−0.2602	0.3186	−0.0379
	(0.72)	(−1.02)	(0.85)	(−0.61)
_cons	2.9303	−2.6470	10.2271	−2.7859**
	(0.13)	(−0.54)	(1.33)	(−2.28)
Individual fixed effects	Yes	Yes	Yes	Yes
Time trend	Yes	Yes	Yes	Yes
N	1224	1224	1224	1020
Wald	360.7653***	377.0282***	942.8933***	565.9782***

Note: The values in parenthesis are *t* statistics

***, **, and * denote significance at the 1%, 5%, and 10% levels, respectively. In the case of corn futures, the risk spillovers from China to the US are insignificant, so this sample is kicked out in the regression.

The results show that in columns (1), (3), and (4), the coefficients of *VIX*_*t*_ are statistically significant at the 10% level. It means that when investor panic is more severe, the intensity of the risk spillover effects is more significant. However, most other variables are insignificant at a 5% significance level. One possible reason is that driving factors of extreme risk spillovers vary over time, leading to the insignificance of these influencing factors when using the overall sample. In particular, the risk diffusion channels may differ between the “stable” (low dependence) and “crisis” (high dependence) regimes. Thus, clarifying the drivers of extreme risk spillovers during crises is imperative.

Next, we detail the risk spillover mechanisms in crises. **Table 14** shows the regression results during the GFC. It indicates statistically significant coefficients for both *VIX*_*t*_ and *Flow*_*USA→CHN*,*t*_ in columns (1) and (2). It indicates that the macro factors and financial shocks significantly influence the risk spillovers from the US to China. The US subprime mortgage crisis resulted in global financial market chaos coupled with the depreciation of the US dollar, and the market panic sharply increased. When market panic intensifies, and concerns about food security escalate, price information from one market can immediately be transmitted to other markets, thus exacerbating the magnitude of risk spillovers. Moreover, massive speculative capital in the international markets moves into international commodity futures markets, such as agricultural commodities [75, 76]. As a result, flows of international capital and a rise in investor panic could accelerate cross-border extreme risk spillovers and endanger a country’s food security. This is a profound lesson from the 2007–2009 GFC, consistent with the conclusions of von Braun and Torero [77].

**Table 14:** The regression results during the GFC.

	(1)	(2)	(3)	(4)
	$\Delta CoVaR_{95\%,t}^{CHN|USA}$	$\Delta CoVaR_{5\%,t}^{CHN|USA}$	$\Delta CoVaR_{95\%,t}^{USA|CHN}$	$\Delta CoVaR_{5\%,t}^{USA|CHN}$
*VIX*_*t*_	0.6766**	−0.0639**	0.0607	−0.0177*
	(2.53)	(−2.48)	(1.62)	(−1.80)
*GEPU*_*t*_	0.0356	0.0006	−0.0041	0.0022
	(0.62)	(0.10)	(−0.55)	(1.10)
*EXR*_*t*_	153.1965	−8.6532	−14.5668	2.7695
	(1.64)	(−0.96)	(−1.34)	(0.95)
*GDP*_*USA*,*t*_	3774.9833***	−197.7799	108.1050	−53.0483
	(2.62)	(−1.42)	(0.64)	(−1.18)
*GDP*_*CHN*,*t*_	−1.79e+03	167.9465	12.3876	13.0501
	(−1.37)	(1.33)	(0.08)	(0.33)
*GPR*_*t*_	−0.4879***	0.0111	−0.0226	0.0068
	(−2.89)	(0.68)	(−1.07)	(1.22)
*TPU*_*USA*,*t*_	−0.0435	−0.0112*		
	(−0.70)	(−1.88)		
*TPU*_*CHN*,*t*_			−0.0007	0.0000
			(−0.15)	(0.03)
*Flow*_*USA→CHN*,*t*_	7.3563**	−0.7807**		
	(2.07)	(−2.27)		
*Flow*_*CHN→USA*,*t*_			0.2830	−0.1540
			(0.76)	(−1.57)
*Pduc*_*USA*,*t*_	−17.4701	−0.1518		
	(−0.77)	(−0.08)		
*Pduc*_*CHN*,*t*_			2.2518	0.0221
			(1.52)	(0.03)
*Biofuel*_*t*_	24.9822	−4.6749	−9.3463	2.3735
	(0.55)	(−1.07)	(−1.62)	(1.55)
_cons	−3.42e+03***	125.0283	24.8971	5.2169
	(−2.74)	(1.04)	(0.16)	(0.13)
Individual fixed effects	Yes	Yes	Yes	Yes
Time trend	Yes	Yes	Yes	Yes
N	126	126	126	105
Wald	263.0215***	1953.1346***	2432.7309***	2238.1920***

Note: The values in parenthesis are *t* statistics

***, **, and * denote significance at the 1%, 5%, and 10% levels, respectively. In the case of corn futures, the risk spillovers from China to the US are insignificant, so this sample is kicked out in the regression.

The coefficients of *GDP*_*USA*,*t*_ and *GPR*_*t*_ are also significant in column (1). It indicates that the US economic downturn, to some extent, has constrained cross-border investment and trade activities, thereby partially restraining the upward risk spillover from the US to China. During the GFC, global geopolitical risk does not experience a sharp change. However, due to measurement issues with this indicator, the financial crisis events have crowded out the newspaper reporting of geopolitical events. This may account for the significance of *GPR*_*t*_ in column (1). Besides, the drivers for risk spillovers from China to the US are all insignificant, except for VIX. This could be attributed to the fact that increased government intervention and policy measures aimed at mitigating the effects of the financial crisis have eclipsed the influence of specific drivers for risk spillovers from China to the US.

**Table 15** presents the results during the US-China trade war. We find the coefficients of *TPU*_*USA*,*t*_/*TPU*_*CHN*,*t*_ are significant in columns (1)–(4). It indicates that the risk spillovers between the US and China are positively related to trade policy uncertainty. The US-China economic and trade interdependence has been growing since 2001. The annual exports of agricultural and related products from the US to China exceed $24 billion [78]. The trade policy uncertainty sharply increases during the US-China trade war, thus affecting the trade imports and intensifying the extreme risk spillovers between them. At this point, the extreme risk spillovers during the US-China trade frictions mainly rely on the impact of trade shocks. These findings highlight the vital role of trade and production shocks in agricultural commodity markets, consistent with Nigatu et al. [62] and Distefano et al. [67]. In addition, macroeconomic factors, financial shocks, supply disruptions, and demand fluctuations offer an explanation for the risk spillovers from China to the US to some extent.

**Table 15:** The regression results during the US-China trade war (only including the period before the COVID-19 pandemic).

	(1)	(2)	(3)	(4)
	$\Delta CoVaR_{95\%,t}^{CHN|USA}$	$\Delta CoVaR_{5\%,t}^{CHN|USA}$	$\Delta CoVaR_{95\%,t}^{USA|CHN}$	$\Delta CoVaR_{5\%,t}^{USA|CHN}$
*VIX*_*t*_	0.0881	−0.0116	0.0385***	−0.0225***
	(0.67)	(−0.26)	(2.89)	(−4.85)
*GEPU*_*t*_	−0.0177	0.0055	−0.0027	0.0028***
	(−1.03)	(0.93)	(−1.34)	(3.97)
*EXR*_*t*_	−3.1446	0.5469	1.6809***	−0.2627*
	(−0.79)	(0.40)	(4.08)	(−1.85)
*GDP*_*USA*,*t*_	−246.5666	−24.4744	228.7875***	−100.4106***
	(−0.44)	(−0.13)	(4.45)	(−5.70)
*GDP*_*CHN*,*t*_	−193.7446	148.8450	−175.4692***	62.0791***
	(−0.35)	(0.79)	(−3.61)	(3.77)
*GPR*_*t*_	0.0173	0.0008	0.0095***	−0.0024**
	(0.58)	(0.08)	(3.18)	(−2.34)
*TPU*_*USA*,*t*_	0.0030**	−0.0011**		
	(2.33)	(−2.44)		
*TPU*_*CHN*,*t*_			0.0008***	−0.0003***
			(4.09)	(−4.65)
*Flow*_*USA→CHN*,*t*_	−1.2639	0.3840		
	(−0.75)	(0.67)		
*Flow*_*CHN→USA*,*t*_			0.7240***	−0.0201
			(2.70)	(−0.22)
*Pduc*_*USA*,*t*_	−14.1964	5.8911		
	(−0.69)	(0.87)		
*Pduc*_*CHN*,*t*_			3.5482***	−1.5894***
			(3.33)	(−2.98)
*Biofuel*_*t*_	−18.3530	1.7167	4.2571***	−1.8464***
	(−1.41)	(0.38)	(3.07)	(−3.91)
_cons	566.9142	−156.6650	−78.3648*	46.0526***
	(1.41)	(−1.14)	(−1.75)	(3.09)
Individual fixed effects	Yes	Yes	Yes	Yes
Time trend	Yes	Yes	Yes	Yes
N	144	144	144	120
Wald	1659.3721***	2462.4512***	2875.1163***	3946.8509***

Note: The values in parenthesis are *t* statistics

***, **, and * denote significance at the 1%, 5%, and 10% levels, respectively. In the case of corn futures, the risk spillovers from China to the US are insignificant, so this sample is kicked out in the regression.

**Table 16** presents the regression results during the period of the COVID-19 pandemic and the Russia-Ukraine 2022 war. In columns (1)–(2), the coefficients of *Pduc*_*USA*,*t*_ are significant, and so are the coefficients of *Flow*_*USA→CHN*,*t*_ at the 10% level. These results demonstrate that international capital flows could also be an important channel for the upside risk spillovers from the US to China, particularly during the period of the COVID-19 pandemic and the Russia-Ukraine 2022 war, which have exacerbated the tensions in international agricultural markets. Significantly, the COVID-19 pandemic and the Russia-Ukraine 2022 war have exacerbated the tensions in international agricultural markets. An escalation of the conflict could trigger investor panic and safe-haven demand, leading to investment funds flowing into agricultural markets, thereby increasing the intensity of market volatility and risk spillovers.

**Table 16:** The regression results during the period of the COVID-19 pandemic and the Russia-Ukraine war in 2022.

	(1)	(2)	(3)	(4)
	$\Delta CoVaR_{95\%,t}^{CHN|USA}$	$\Delta CoVaR_{5\%,t}^{CHN|USA}$	$\Delta CoVaR_{95\%,t}^{USA|CHN}$	$\Delta CoVaR_{5\%,t}^{USA|CHN}$
*VIX*_*t*_	−0.0052	0.0059	−0.0072	0.0011
	(−0.18)	(0.59)	(−0.56)	(0.33)
*GEPU*_*t*_	−0.0006	−0.0016	0.0069***	−0.0012**
	(−0.12)	(−0.90)	(3.02)	(−1.97)
*EXR*_*t*_	0.2586	−0.1364	−1.4683**	0.3550*
	(0.18)	(−0.26)	(−2.22)	(1.93)
*GDP*_*USA*,*t*_	−20.8412	3.4564	−0.9938	0.1139
	(−0.85)	(0.40)	(−0.11)	(0.05)
*GDP*_*CHN*,*t*_	−15.0068**	7.8043***	−1.8198	0.4806
	(−2.07)	(3.09)	(−0.59)	(0.59)
*GPR*_*t*_	0.0038	−0.0024	0.0048**	−0.0012*
	(0.73)	(−1.28)	(2.03)	(−1.93)
*TPU*_*USA*,*t*_	−0.0011	0.0006		
	(−0.52)	(0.82)		
*TPU*_*CHN*,*t*_			−0.0011***	0.0003***
			(−3.10)	(2.58)
*Flow*_*USA→CHN*,*t*_	0.8456*	−0.3083*		
	(1.76)	(−1.86)		
*Flow*_*CHN→USA*,*t*_			0.1784	−0.0503
			(0.77)	(−0.82)
*Pduc*_*USA*,*t*_	−5.9262***	0.7165***		
	(−2.91)	(2.90)		
*Pduc*_*CHN*,*t*_			1.6332	−0.1404
			(0.98)	(−0.90)
*Biofuel*_*t*_	5.3511	−1.6776	−0.1820	0.2539
	(1.62)	(−1.41)	(−0.12)	(0.63)
_cons	47.4816*	−15.8976*	14.2454	−3.9018
	(1.74)	(−1.65)	(1.16)	(−1.24)
Individual fixed effects	Yes	Yes	Yes	Yes
Time trend	Yes	Yes	Yes	Yes
N	216	216	216	180
Wald	832.0097***	881.5019***	468.3347***	209.2848***

Note: The values in parenthesis are *t* statistics

***, **, and * denote significance at the 1%, 5%, and 10% levels, respectively. In the case of corn futures, the risk spillovers from China to the US are insignificant, so this sample is kicked out in the regression.

Furthermore, the risk spillovers from the US to China also rely heavily on supply-side shocks. Production and supply chain shortages due to blockades, travel restrictions, and quarantines after the outbreak of the COVID-19 pandemic have reduced the availability of food [79–81]. This exacerbates the fluctuations and risk spillovers in these two countries’ markets. Therefore, during the COVID-19 pandemic and the Russia-Ukraine war in 2022, the risk spillovers from the US to China highlight the supply-side shocks (the non-financial channel). These findings are also in line with the ideas of Laborde et al. [82]. Besides, macro factors and trade shocks have had a significant impact on risk spillovers from China to the US.

In summary, the lessons from the GFC do not apply to the current period of the COVID-19 pandemic. As for the risk spillovers from the US to China, the GFC highlights the financial channel. The significant volatility in the agricultural futures markets can primarily be attributed to the financialization of commodities. However, after the outbreak of COVID-19, the economic isolation and pause triggered short-term disruptions and interruptions in production and trade chains. The extreme risk spillovers from the US to China during the COVID-19 pandemic rely mainly on supply-related shocks (the non-financial channel). Besides, despite the risk spillover mechanisms from China to the US being confused, it is worth emphasizing that macro factors play an important role in driving shock transmissions.

## 6. Summary, conclusion and recommendation

This paper captures the extreme risk spillovers between the US and Chinese agricultural commodity futures markets during significant crises using a copula-CoVaR model. Moreover, by incorporating them into a Markov regime-switching framework, we identify a shift in dependence structure during normal (low-dependence) and crisis (high-dependence) times. Finally, through the analysis of extreme risk spillovers with dependence switching, this research reveals their underlying drivers, particularly within the contexts of the GFC, the US-China trade war, and the period of the COVID-19 pandemic and the Russia-Ukraine 2022 war.

We summarize several findings. First, we find significant and asymmetric spillovers of extreme risk between the US and Chinese markets. Notably, these markets are more likely to boom than to crash together, highlighting the prevention of a food crisis caused by cross-border upside risk spillovers. Second, the dependence structure between these markets shows two distinct regimes in times of stability and crisis. The extreme risk spillover effects are highly volatile and are significantly intensified by the GFC, COVID-19, and the Russia-Ukraine war. Third, our findings support the role of financial, trade, and supply-side shocks in driving the risk spillovers from the US to Chinese markets. For example, during the GFC, international capital flows into Chinese markets from the US (the financial channel) exacerbate the dissemination of risk from the US to China. The trade shocks account for the changes in extreme risk spillovers during the US-China trade war. By contrast, the COVID-19 pandemic and conflict in Ukraine highlight the role of supply-side shocks (the non-financial channel). In addition, macro factors are also essential in driving risk contagion.

The recommendations arising from these findings are as follows: For investors, this paper highlights the interconnectivity of agricultural futures markets between the US and China. The increasing financialization of commodities has led to severe and frequent fluctuations in the international agricultural futures markets. In this context, investors in the US and China should anticipate the impact of fluctuations in the other markets on their domestic markets. They can build up dynamic risk monitoring/early warning mechanisms for their portfolios and seek diversified protection for their agricultural commodity investments. Furthermore, investors should respond to trade, supply, and financial shocks arising from local and global extreme risk events in a timely manner and adjust their asset portfolios promptly.

For policymakers, they should strengthen their risk monitoring frameworks to detect early signs of extreme risk spillovers between US and Chinese markets. Both countries should develop and maintain crisis preparedness plans for their agricultural sectors, including strategies for managing sudden shifts in market dependence structures. Moreover, stricter financial regulations should be strengthened to avoid the large-scale hot capital flows into the agricultural commodity markets and the investors’ excess speculation, thus avoiding the sharp fluctuations in food markets and the significant threat to food security. In the face of production or trade distribution shocks, agricultural price support or stabilization policies should also be introduced to stabilize the agricultural futures markets for both upside and downside distress. Finally, our study also highlights the cooperation of countries/regions to address the excess fluctuations in food markets, such as by refraining from food trade protectionism.

This paper is limited to focusing on the extreme risk spillovers between China and the US. In the future, it may be exciting follow-up work if scholars investigate cross-border extreme risk spillovers using a global sample and analyze systemic risk spillovers in the global food system. It will help to provide a comprehensive understanding of how risks propagate across different regions and markets and offer insights into the interconnectedness and vulnerabilities of the global food supply chain. Moreover, the impact of extreme risk spillovers in the international food markets on global food security could be shed more light on. This line of research could lead to the development of measures and policies to safeguard food security in the face of systemic risk and extreme events in the international food markets.

## Supporting information

S1 Data (ZIP)
